# Distribution Analysis of Hydrogenases in Surface Waters of Marine and Freshwater Environments

**DOI:** 10.1371/journal.pone.0013846

**Published:** 2010-11-05

**Authors:** Martin Barz, Christian Beimgraben, Torsten Staller, Frauke Germer, Friederike Opitz, Claudia Marquardt, Christoph Schwarz, Kirstin Gutekunst, Klaus Heinrich Vanselow, Ruth Schmitz, Julie LaRoche, Rüdiger Schulz, Jens Appel

**Affiliations:** 1 Botanisches Institut, Christian-Albrechts-Universität, Kiel, Germany; 2 Forschungs- und Technologiezentrum Westküste (FTZ) der Christian-Albrechts-Universität, Büsum, Germany; 3 School of Life Sciences, Arizona State University, Tempe, Arizona, United States of America; 4 Institut für Allgemeine Mikrobiologie, Christian-Albrechts-Universität, Kiel, Germany; 5 Leibniz-Institute of Marine Sciences, IFM-GEOMAR, Kiel, Germany; Universidad Miguel Hernandez, Spain

## Abstract

**Background:**

Surface waters of aquatic environments have been shown to both evolve and consume hydrogen and the ocean is estimated to be the principal natural source. In some marine habitats, H_2_ evolution and uptake are clearly due to biological activity, while contributions of abiotic sources must be considered in others. Until now the only known biological process involved in H_2_ metabolism in marine environments is nitrogen fixation.

**Principal Findings:**

We analyzed marine and freshwater environments for the presence and distribution of genes of all known hydrogenases, the enzymes involved in biological hydrogen turnover. The total genomes and the available marine metagenome datasets were searched for hydrogenase sequences. Furthermore, we isolated DNA from samples from the North Atlantic, Mediterranean Sea, North Sea, Baltic Sea, and two fresh water lakes and amplified and sequenced part of the gene encoding the bidirectional NAD(P)-linked hydrogenase. In 21% of all marine heterotrophic bacterial genomes from surface waters, one or several hydrogenase genes were found, with the membrane-bound H_2_ uptake hydrogenase being the most widespread. A clear bias of hydrogenases to environments with terrestrial influence was found. This is exemplified by the cyanobacterial bidirectional NAD(P)-linked hydrogenase that was found in freshwater and coastal areas but not in the open ocean.

**Significance:**

This study shows that hydrogenases are surprisingly abundant in marine environments. Due to its ecological distribution the primary function of the bidirectional NAD(P)-linked hydrogenase seems to be fermentative hydrogen evolution. Moreover, our data suggests that marine surface waters could be an interesting source of oxygen-resistant uptake hydrogenases. The respective genes occur in coastal as well as open ocean habitats and we presume that they are used as additional energy scavenging devices in otherwise nutrient limited environments. The membrane-bound H_2_-evolving hydrogenases might be useful as marker for bacteria living inside of marine snow particles.

## Introduction

The composition of earth's atmosphere is the result of a number of concurring processes and a matter of continuous change. Especially the amount of trace gases governs important aspects of the gas cover of our planet, such as its retention capacity of heat or the amount of ozone present. After methane, hydrogen is the second most abundant trace gas in the atmosphere, making up around 0.5 ppm to 0.6 ppm [1], [2].

Approximately 90% of hydrogen evolution is due to photochemical oxidation of hydrocarbons such as methane in the atmosphere, the combustion of fossil fuels and biomass burning. Natural evolution results from volcanic activity, the nitrogen fixation process in legumes and an uncharacterized source in the oceans. The latter comprises the majority with around 6% (6 Tg per year [3]).

The removal of hydrogen is either due to its reaction with hydroxyl radicals in the atmosphere or by its reaction with hydrogenases in the soil. In particular, hydrogen uptake into the soil is responsible for the largest term with an estimated 75% to 77% globally [1]–[5]. This is further corroborated by the lower average concentration of hydrogen found on the northern hemisphere, with its larger landmass [1]. Hydrogen uptake was attributed to aerobic hydrogen-oxidizing bacteria and extracellular enzymatic activity. Abiotic removal has been previously considered since hydrogen concentrations are below the threshold level found for cultures of aerobic hydrogen oxidizing bacteria that still maintains growth [6].

In contrast to soil, supersaturating concentrations of hydrogen have been measured in aquatic environments. In all cases, concentrations were highest at the surface and steeply decreased down to the thermocline while the deep ocean is undersaturated. Although a systematic analysis is not available it appears that surface waters of tropical and subtropical oceans are generally hydrogen sources [7]–[9]. In contrast, concentrations lower than the expected atmospheric equilibrium have been observed in higher latitudes and both hydrogen uptake and production vary depending on the season [10], [11]. In some fresh water lakes supersaturation has also been found [12], with a maximum at dawn [13]. The highest hydrogen concentrations were in the upper water column, which correlated with the maximum of primary production [13], [14].

Marine hydrogen uptake has been attributed to particulate fractions of 0.2 µm to 5 µm in size [11] and, like in freshwater lakes, most probably correlates with aerobic hydrogen-oxidizing bacteria [13]. Hydrogen production in the oceans was found to depend on solar radiation and clearly shows a diurnal variation with a maximum around noon [8], [9]. Since the nitrogenase inevitably produces at least one molecule of hydrogen per dinitrogen reduced to ammonia, cyanobacterial nitrogen fixation is thought to be the major source of hydrogen in these oceanic regions. Studies on heterocystous cyanobacteria demonstrated that hydrogen cycling by these strains is highly effective, although under CO_2_-limitation around 0.1 nmol H_2_ h^−1^ (mg chlorophyll)^−1^ escapes to the environment [15]. In contrast to this, in-situ measurements of *Trichodesmium thiebautii* (former *Oscillatoria thiebautii*), which is one of the major oceanic N-fixing strains, questioned whether its hydrogen evolution is actually sufficient to explain the concentrations found [16].

Recently it was shown that photochemical production of hydrogen from chromogenic dissolved organic matter can contribute, at least in part, to hydrogen production in fresh water lakes as well as coastal seawater [17]. Therefore, abiotic sources should be taken into account.

In the microbial world hydrogen is a valuable energy source that is exchanged efficiently between different prokaryotes and anaerobic eukaryotes. Some produce hydrogen while fermenting whereas others capture it to drive anaerobic or aerobic respiration and make use of its energy. A wealth of different enzymes called hydrogenases have been found in microorganisms that are able to split or form hydrogen [18], [19].

Hydrogenases are classified according to their metal content into the Fe-, FeFe-, and NiFe-varieties. Fe-hydrogenases are confined to the methanogenic archaea and FeFe-hydrogenases occur in bacteria and anaerobic eukaryotes. NiFe-hydrogenases are separated into 4 different groups and are widespread in archaea and bacteria [19], [20]. Most purified hydrogenases are only active under anoxic conditions, but there are some NiFe-hydrogenases from aerobic H_2_-oxidizing bacteria that are able to oxidize hydrogen at ambient oxygen concentrations [21].

Although hydrogenases have been investigated for a long time in a variety of different microorganisms it is rather difficult to deduce their physiological function on the basis of their classification alone. In Table 1 a tentative assignment of their metabolic roles is given. However, this assignment needs to be treated cautiously since several studies found surprising variations. Hydrogenase 2 of *E. coli* belongs to the group 1 H_2_-uptake hydrogenases and was originally described as H_2_-oxidizing enzyme [22]. In contrast, recent electrochemical data suggests that the hydrogenase 2 is working as a bidirectional enzyme [23]. Another interesting variance was found in case of the group 4 membrane-bound H_2_-evolving hydrogenase. In many cases these enzymes seem to be used under fermentative conditions to generate a proton gradient (e.g. [24]) but in other cases they might be used to oxidize H_2_ and reduce ferredoxin with the concomitant use of a proton gradient [25] or even for H_2_ uptake in N-fixing bacteria [26].

**Table 1:** Overview of all the known hydrogenase enzymes.

Group	Name	Tentative function	O_2_ resistance
**Fe-hydrogenase**			
One Group	Hmd hydrogenase	Occurs only in methanogens and is used for H_2_-uptake during methanogenesis	its cofactor is sensitive against oxygen
**FeFe-hydrogenases**			
No groups assigned yet	Periplasmic and cytoplasmic enzymes	Periplasmic enzymes are probably H_2_-oxidizing whereas cytoplasmic enzymes are H_2_-evolving	No resistant enzymes known, rapid inactivation by O_2_
**NiFe-hydrogenases**			
1	Membrane-bound H_2_-uptake hydrogenases	H_2_ uptake under anaerobic and aerobic conditions	Some resistant enzymes known
2a	Cyanobacterial uptake hydrogenases	H_2_ uptake under N_2_-fixing conditions	No resistant enzymes known
2b	H_2_-sensing hydrogenases	H_2_ receptor that activates the expression of hydrogenase structural genes	Resistant
3a	F_420_-reducing hydrogenases	H_2_ uptake during methanogenesis	No resistant enzymes known
3b	Bifunctional NAD(P) hydrogenases	Function unknown	No resistant enzymes known
3c	Methyl-viologen-reducing hydrogenases	H_2_ uptake during methanogenesis	No resistant enzymes known
3d	Bidirectional NAD(P)-linked hydrogenases	H_2_ uptake for the generation of NAD(P)H or H_2_ evolution	Some resistant enzymes known
4	Membrane-bound H_2_-evolving hydrogenases	H_2_ evolution under fermentative conditions in some bacteria and H_2_ uptake for the reduction of ferredoxin in others, both processes are either accompanied by a proton gradient formation or the use of a proton gradient for reverse electron transfer	No resistant enzymes known

For all the different classes [19], [20] a tentative function is given.

Systematic studies concerning the distribution of hydrogenases in different habitats to unravel their ecophysiological role are not yet available. Apart from the investigation of some specific soil hydrogenases [27], [28] only two studies attempted the amplification of FeFe-hydrogenase sequences from microbial mats [29], [30]. Although these works showed a surprising variety of these hydrogenases the short sequences amplified preclude any assignment of their function.

The hydrogen concentrations found in a variety of surface waters prompted us to investigate the presence and distribution of all known hydrogenases in marine and freshwater environments. Moreover, the ecological distribution of their genes was analyzed to collect valuable hints for their physiological functions and their oxygen tolerance.

To this end we analyzed the distribution of hydrogenases in cyanobacteria since they are one of the largest prokaryotic groups that occur in aquatic surface waters. The search was then expanded to the complete genomes of bacteria isolated from marine surface waters (http://www.ncbi.nlm.nih.gov/sutils/genom_table.cgi [31]) and the global ocean sampling metagenomic database (http://camera.calit2.net/)[32]–[34] for all the families of hydrogenases as classified by Vignais et al. [20] and Vignais and Billoud [19]. In parallel, we investigated DNA isolated from samples taken from the North Atlantic, Mediterranean Sea, North Sea, the Baltic Sea and the fresh water lakes Westensee and Selenter See in Northern Germany for the presence of the genes of the bidirectional NAD(P)-linked hydrogenase. Our results reveal that these enzymes are surprisingly widespread in surface waters and a clear bias toward waters with terrestrial influence is obvious.

## Results

### Distribution of hydrogenases in cyanobacterial genomes

Cyanobacteria are known to harbor two different NiFe-hydrogenases. One is called bidirectional (group 3d) since it can produce or take up hydrogen, depending on the physiological conditions and the other is an uptake hydrogenase (group 2a) that is linked to the nitrogen fixation process and seems to be confined to diazotrophic strains [35], [36]. A phylogenetic analysis revealed a close ancestry of both hydrogenases to the filamentous anoxygenic photosynthetic bacteria (the former green non-sulfur bacteria)[37].

**Figure 1:**
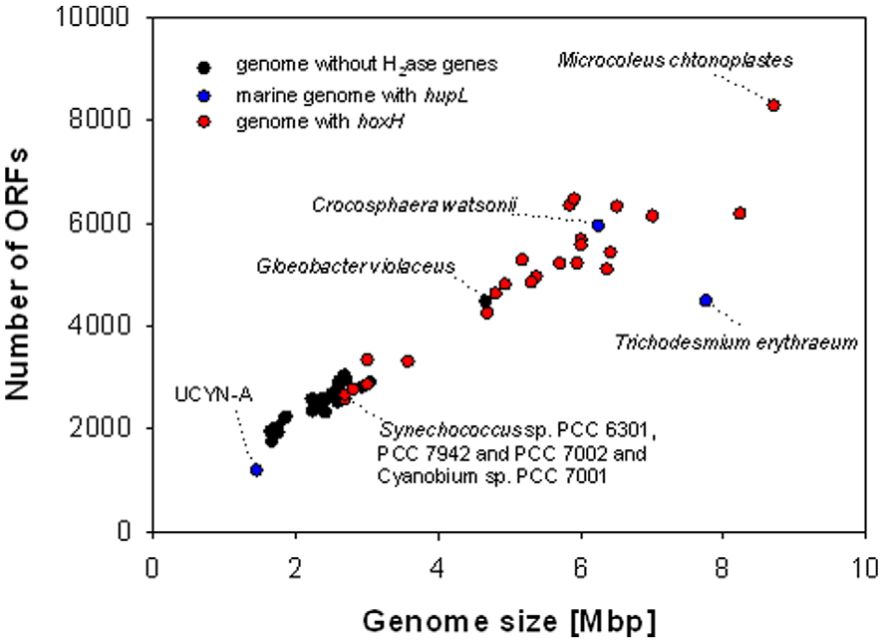
Comparison of cyanobacterial genome sizes and the distribution of the bidirectional NAD(P) linked hydrogenase gene *hoxH*. Genomes without the bidirectional hydrogenase are depicted in black and those with it are red. The marine diazotrophic cyanobacteria containing the genes of the uptake hydrogenase *hupL* are shown in cyan. The cluster of black circles at the lower left end of the line represents the small genomes of the *Prochlorococcus* and *Synechococcus* strains.

A search of genebank (http://www.ncbi.nlm.nih.gov/) and cyanobase (http://bacteria.kazusa.or.jp/cyanobase/) for all available cyanobacterial sequences revealed the presence of the bidirectional NAD(P)-linked hydrogenase (the large subunit HoxH was used in the BLAST search [38]) in all the freshwater strains and all the strains isolated from microbial mats, salt marshes, and the intertidal zone (Table 2). In contrast, only four out of the seven available coastal genomes harbor the gene for the bidirectional enzyme and it was completely absent in oceanic strains. Genomestreamlining and iron limitation [39] in the open ocean could be used as arguments for the absence of the bidirectional hydrogenase genes in the picoplanktonic *Prochlorococcus* and *Synechococcus* strains. But even the typical open ocean strains *Crocosphaera watsonii* and *Trichodesmium erythraeum* with genome sizes above 6 Mbp do not harbor this hydrogenase, although both have the uptake hydrogenase, which has an iron requirement similar to the bidirectional enzyme (Fig. 1 and Table 2). In addition the unicellular marine strain UCYN-A that lacks photosystem II shows an extremely reduced genome and still contains the *hup*-genes [40].

**Table 2:** Occurrence of the bidirectional NAD(P)-linked hydrogenase (HoxH) and the membrane-bound uptake hydrogenase (HupL) in cyanobacteria.

Strain	Environ ment	size	HoxH^a^	NifJ	HupL	NifD
***Anabaena variabilis*** ** ATCC 29413**	freshwater	6.36	YP_325153	YP_323551 YP_321599	YP_325087	YP_324742
*Arthrospira maxima* CS-328	freshwater	6.0	ZP_03273562	ZP_03273569		
*Cylindrospermopsis raciborskii* CS-505	freshwater		ZP_06307638	ZP_06307770 ZP_06309351	ZP_06309263	ZP_06309411
*Gloeocapsa alpicola* str. CALU 743	freshwater		AAO85440			
***Microcystis aeruginosa*** ** NIES-843**	freshwater	5.84	YP_001656435	YP_001658828		
***Microcystis aeruginosa*** ** PCC 7806**	freshwater	5.17	CAO89286	CAO88863		
***Nostoc*** **sp. PCC 7120**	freshwater	6.41	NP_484809	NP_485951 NP_486843	NP_484720	NP_485484
*Prochlorothrix hollandica*	freshwater		U88400			
*Raphidiopsis brookii* D9	freshwater		ZP_06303751	ZP_06305273		
***Synechococcus elongatus*** ** PCC 6301**	freshwater	2.69	YP_172265	YP_172431		
***Synechococcus elongatus*** ** PCC 7942**	freshwater	2.69	YP_401572	YP_401401		
***Synechocystis*** ** sp. PCC 6803**	freshwater	3.57	NP_441411	NP_442703		
*Anabaena siamensis* TISTR 8012	rice field		AAN65267		AAN65266	ABA02237
***Cyanothece*** ** sp. PCC 7424**	rice field	5.94	ZP_02972728	YP_002376576	ZP_02973433	YP_002377414
***Cyanothece*** ** sp. PCC 7425** b	rice field	5.37	YP_002484718	YP_002485040		ZP_03139427
*Cyanothece* sp. PCC 7822	rice field	5.7	ZP_03154336	ZP_03157112	ZP_03153783	ZP_03154128
***Cyanothece*** ** sp. PCC 8801**	rice field	4.68	ZP_02942892	YP_002374020	ZP_02941033	ZP_02943179
***Cyanothece*** ** sp. PCC 8802**	rice field	4.8	ZP_03143669	ZP_03141892	ZP_03142797|	ZP_03144923|
*Arthrospira maxima* FACHBSM	saline marsh		AAQ63961			
*Arthrospira platensis* FACHB341	saline marsh		AAQ63964			
*Arthrospira platensis* FACHB439	saline marsh		AAQ63960			
*Arthrospira platensis* FACHB440	saline marsh		AAQ63963			
*Arthrospira platensis* FACHB791	saline marsh		AAQ91344			
*Arthrospira platensis* FACHBOUQDS6	saline marsh		AAQ63959			
*Microcoleus chthonoplastes* PCC 7420	saline marsh	8.67	YP_002619903	YP_002620835		ZP_05024116
*Lyngbya aestuarii* CCY 9616	marine microbial mat	7.0	ABD34839		ABD34838	ABD34836^c^
*Lyngbya majuscula* CCAP 1446/4	marine microbial mat		AY536043		AAO66476	AAY78884
*Lyngbya* sp. PCC 8106	marine microbial mat	7.0	ZP_01622077	ZP_01622083	ZP_01619041	ZP_01620767
*Cyanobium s*p. PCC 7001	intertidal zone	2.8	YP_002597857	YP_002597848		
***Cyanothece*** ** sp. ATCC 51142**	intertidal zone	4.93	YP_001803731	YP_001802370	YP_001802481	YP_001801977
***Synechococcus*** ** sp. PCC 7002**	intertidal zone	3.00	YP_001733469	YP_001734690		
*Synechococcus* sp. PCC 7335	intertidal zone	6.0	YP_002710310	YP_002711016 YP_002710302		YP_002711054
*Cyanothece* sp. CCY 0110	coastal	5.9	ZP_01727423	ZP_01730229	ZP_01728928	ZP_01727766
*Nodularia spumigena* CCY 9414	coastal	5.3	ZP_01629499	ZP_01630855	ZP_01628406	ZP_01628430
*Spirulina subsalsa* FACHB351	coastal		AY345592			
***Synechococcus*** ** sp. CC9605**	coastal	2.51				
***Synechococcus*** ** sp. CC9902**	coastal	2.23				
*Synechococcus* sp. BL107	coastal	2.3				
*Synechococcus* sp. WH 5701	coastal	3.0	ZP_01085930	ZP_01085923		
***Crocosphaera watsonii*** ** WH 8501**	open ocean	6.24		ZP_00518015	ZP_00519188	ZP_00516387
***Prochlorococcus marinus*** ** str. AS9601**	open ocean	1.67				
***Prochlorococcus marinus*** ** str. MIT 9211**	open ocean	1.69				
***Prochlorococcus marinus*** ** str. MIT 9215**	open ocean	1.74				
***Prochlorococcus marinus*** ** str. MIT 9301**	open ocean	1.64				
***Prochlorococcus marinus*** ** str. MIT 9303**	open ocean	2.68				
***Prochlorococcus marinus*** ** str. MIT 9312**	open ocean	1.70				
***Prochlorococcus marinus*** ** str. MIT 9313**	open ocean	2.41				
***Prochlorococcus marinus*** ** str. MIT 9515**	open ocean	1.70				
***Prochlorococcus marinus*** ** str. NATL1A**	open ocean	1.86				
***Prochlorococcus marinus*** ** str. NATL2A**	open ocean	1.84				
***Prochlorococcus marinus subsp. marinus*** ** str. CCMP1375**	open ocean	1.75				
***Prochlorococcus marinus subsp. pastoris*** ** str. CCMP1986**	open ocean	1.75				
***Synechococcus*** ** sp. CC9311**	open ocean	2.61				
***Synechococcus*** ** sp. WH 7803**	open ocean	2.37				
*Synechococcus* sp. WH 7805	open ocean	2.6				
***Synechococcus*** ** sp. WH 8102**	open ocean	2.43				
***Trichodesmium erythraeum I*** **MS101**	open ocean	7.75			YP_722943	YP_723618
Cyanobacterium UCYN-A	open ocean	1.44			YP_003421184	YP_003421697
***Synechococcus*** ** sp. RCC307**	Mediterranean Sea	2.22				
*Synechococcus* sp. RS9916	Red Sea	2.7				
*Synechococcus* sp. RS9917	Red Sea	2.6				
***Synechococcus*** ** sp. JA-2-3B'a(2-13)**	hot spring	3.04				YP_476681
***Synechococcus*** ** sp. JA-3-3Ab**	hot spring	2.93				YP_475237
***Thermosynechococcus elongatus*** ** BP-1**	hot spring	2.59				
***Acaryochloris marina***	ascidian	6.50	YP_001521996	YP_001522063		
*Arthrospira platensis* str. Paraca	Salt lake		ZP_06307638	ZP_06381891		
***Gloeobacter violaceus*** ** sp. PCC 7421**	rock	4.66				
*Nostoc azollae*	Symbiont with water fern		ZP_03765204		ZP_03768004	ZP_03768758
***Nostoc punctiforme*** ** sp. PCC 73102**	symbiont with cycad	8.23		YP_001867453	ZP_00112356	ZP_00112319
*Nostoc* sp. PCC 7422	symbiont with cycad	∼10	BAE46796		BAE46791	

a the genomes have been searched by using the respective protein sequences.

b Cyanothece sp. PCC 7425 is the only cyanobacterium with the gene of a bifunctional (NADP) hydrogenase (YP_002483374).

The 69 strains have been separated according to the habitat they have been isolated from. *Leptolyngbya valderiana* BDU 20041 has been omitted from the analysis although it is provided in the genebank (http://www.ncbi.nlm.nih.gov/sutils/genom_table.cgi) since only 89 kbp of its genome has been sequenced. The presence of NifD is given as a marker for the nitrogenase. Completely sequenced strains are given in bold.

All the completely sequenced cyanobacterial strains that harbor the bidirectional hydrogenase genes also harbor the gene of a pyruvate:flavodoxin/ferredoxin oxidoreductase (PFOR), *nifJ*. In two genomes (*Synechococcus* WH 5701 and *Arthrospira maxima*), this gene is either part of the *hyp*-gene cluster or in close proximity to the *hox*-genes, suggesting that the birdirectional hydrogenase is used to dispose of electrons during fermentation via a PFOR-like enzyme (Table 2).

The occurrence of the uptake hydrogenase (HupL, group 2a) in cyanobacteria does not correlate with a specific habitat but with the diazotrophy of the respective strains, as indicated by the presence of the nitrogenase genes (e.g. NifD)(Table 2). Of the completely sequenced genomes two *Synechcococcus* strains isolated from a hot spring and *Cyanothece* sp. PCC 7425 harbor the nitrogenase genes but no uptake hydrogenase. This confirms the previous finding of a marine nitrogen-fixing *Synechococcus* strain without an uptake hydrogenase [37].

*Cyanothece* sp. PCC 7425 is the only strain containing the genes of the bifunctional NAD(P) linked hydrogenase (group 3b)(Table 2) but expression and metabolic activity of this enzyme have not yet been demonstrated.

### Distribution of hydrogenases in genomes of heterotrophic bacteria isolated from marine surface waters

Representatives of each of the hydrogenase classes were used to search the completely sequenced prokaryotic genomes in the genebank (Table 3). Of the approximately 1210 prokaryotic genomes (as of March 2010) 149 were isolated from marine surface waters and in 33 of these genomes, one or several hydrogenases occur, making up 22% of the total (Table 4, Table S1 supporting information). Since a number of the analyzed genomes is still not complete, this proportion is a minimum estimate. If divided into coastal and open ocean isolates, 25% of the coastal and 14% of the open ocean strains have hydrogenase genes.

**Table 3:** Hydrogenase and HypX sequences used for searches of the completely sequenced genomes and the GOS metagenomic database.

Hydrogenase	Organism	Accession number
Fe-hydrogenase	*Methanocaldococcus jannschii*	Q58194
FeFe-hydrogenase	*Clostridium pasteurianum*	P29166
NiFe-hydrogenase group 1 Membrane-bound H_2_ uptake	*Desulfovibrio vulgaris*	P21852
NiFe-hydrogenase group 2a Cyanobacterial uptake	*Nostoc* sp. PCC 7120	NP_484720
NiFe-hydrogenase group 2b H_2_-Sensing	*Ralstonia eutropha*	NP_942663
NiFe-hydrogenase group 3a F_420_-reducing	*Methanocaldococcus jannschii*	Q60338
NiFe-hydrogenase group 3b Bifunctional NAD(P) linked	*Chlorobium tepidum* TLS	NP_662771
NiFe-hydrogenase group 3c MV-reducing	*Methanococcus voltae*	ZP_02193988
NiFe-hydrogenase group 3d Bidirectional NAD(P) linked	*Synechocystis* sp. PCC 6803	BAA18091
NiFe-hydrogenase group 4 Membrane-bound H_2_-evolving	*Escherichia coli*	NP_417201
NiFe-hydrogenase maturation protein HypX	*Ralstonia eutropha*	NP_942660

The hydrogenases were classified according to Vignais et al. 2001 [20].

**Table 4:** Marine bacteria with FeFe-hydrogenases and NiFe-hydrogenases of the different classes.

Coastal/open ocean	strain	FeFe	group 1	group 2a	group2b	group 3a	group 3b	group 3c	group 3d	group 4	HypX
	**Actinobacteria**										
**C**	*Mycobacterium marinum* M		YP_001850173				YP_001851771				
**O**	*Rhodococcus erythropolis* PR4		YP_002766098				YP_002766851				
	**Bacteroidetes/Chlorobi**										
**C**	*Flavobacteria bacterium* MS024-2A		ZP_03702421								
**C**	*Prosthecochloris aestuarii* DSM 271		YP_002015547				YP_002016588				
**O**	*Robiginitalea biformata* HTCC2501		ZP_01119574								
	**Mollicutes/others**										
**C**	Candidatus *Koribacter versatilis* Ellin345		YP_593314								
**C**	*Planctomyces maris* DSM 8797						ZP_01852867				
**C**	*Verrucomicrobiae bacterium* DG1235						YP_002715357				
	**Proteobacteria**										
**C**	*Magnetococcus sp.* MC-1		YP_866409		YP_866399		YP_864809				
	**α-Proteobacteria**										
**C**	*Labrenzia aggregata* IAM 12614		ZP_01550392		ZP_01550270				ZP_01545563		
**C**	*Labrenzia alexandrii* DFL-11								YP_002610401		
**O**	*Roseovarius sp.* HTCC2601		ZP_01443057		ZP_01443054						
**C**	*Roseovarius sp.* TM1035		ZP_01881109		ZP_01881113						
**C**	*Sagittula stellata* E-37		ZP_01748533		ZP_01748530						
**O**	*Sphingopyxis alaskensis* RB2256			YP_611130							
	**δ-Proteobacteria**										
**C**	*Hahella chejuensis* KCTC 2396								YP_431451		
**O**	*Neptuniibacter caesariensis*		ZP_01166595	ZP_01167270	ZP_01166020						ZP_01166042
**C**	*Psychromonas ingrahamii* 37								YP_942646		YP_942640
**C**	*Shewanella baltica* OS155		YP_001050263								
**C**	*Shewanella baltica* OS185		YP_001366120								
**C**	*Shewanella baltica* OS195		YP_001554352								
**C**	*Shewanella baltica* OS223		YP_002358323								
**C**	*Shewanella frigidimarina* NCIMB 400		YP_750788								
**C**	*Shewanella putrefaciens* CN-32		YP_001183609								
**C**	*Shewanella sp.* ANA-3	YP_868355	YP_869516								
**C**	*Shewanella sp.* MR-4	YP_735375	YP_733952								
**C**	*Shewanella sp.* MR-7		YP_738201								
**C**	*Shewanella sp.* W3-18-1		YP_963312								
**C**	*gamma proteobacterium* NOR51-B						YP_002656756				
	**Vibrionaceae**										
**C**	*Photobacterium profundum* 3TCK									ZP_01218749	
**O**	*Photobacterium sp.* SKA34		ZP_01160131							ZP_01161272	
**C**	*Vibrio angustum* S14		ZP_01234606							ZP_01234036	

The presence of HypX, an accessory gene responsible for oxygen tolerance of the soluble hydrogenase of *Ralstonia eutropha,* was included in the search.

The genomes of two *Shewanella* strains (ANA-3 and MR-4) have all the genes necessary for the expression of a FeFe-hydrogenase. Since this type of hydrogenase is extremely sensitive against and irreversibly inactivated by oxygen [41], this is a surprising finding. However, it should be noted that one strain (ANA-3) has been isolated from a wooden pier that might have been occupied by biofilms that could become anaerobic and the other strain (MR-4) was isolated from the Black Sea, which is the world largest anoxic basin [42]. Therefore, both are considered exceptions and will not be discussed any further.

Concerning the NiFe-hydrogenases, there are 24 genomes with a membrane-bound H_2_-uptake hydrogenase (group 1), two genomes with a cyanobacterial-type uptake hydrogenase (group 2a)(*Sphingopyxis alaskensis* RB2256 and *Neptuniibacter caesariensis*), six genomes with a sensor hydrogenase (group 2b), seven genomes with a bifunctional hydrogenase (group 3b), four genomes with a bidirectional NAD(P)-linked hydrogenase (group 3d), and three genomes with a membrane-bound H_2_-evolving hydrogenase (group 4) similar to hydrogenase 3 of *E. coli*.

The genomes of the *Roseovarius* group contain large gene clusters with the membrane-bound hydrogenase in conjunction with a sensor hydrogenase and the whole complement of the two-component system (Fig. S1, supporting information). The sensor enzyme is a receptor that enables the cells to detect hydrogen in the environment and to activate transcription of the hydrogenase structural genes [43]–[46]. The same gene clusters also contain a number of additional genes that encode for proteins such as HupK that have been shown to be necessary for the production of an oxygen tolerant hydrogenase in *R. eutropha* [47], [48].

The genomes of the *Vibrionaceae* harbor a membrane-bound H_2_-evolving hydrogenase (Fig. S2, supporting information) and a second membrane-bound hydrogenase. This is the necessary combination that can be used under anaerobic conditions to establish a proton gradient by hydrogen cycling in a single cell [49].

Additionally, the genome of *N. caesariensis* (former *Oceanospirillum* [50]) is worth mentioning. It contains a membrane-bound enzyme, a cyanobacterial like uptake hydrogenase, a sensor, and a bifunctional hydrogenase. A phylogenetic analysis confirmed that the HypX encoded in its genome belongs to the group of hydrogenase maturation factors (Fig. S3, supporting information). HypX was shown to render the soluble hydrogenase of the Knallgas bacterium *Ralstonia eutropha* oxygen insensitive [51]. The membrane-bound hydrogenase of *N. caesariensis* is a close relative of the same hydrogenase of *R. eutropha* (Fig. S4, supporting information), which is evidence that this bacterium and the *Roseovarius* strains are able to perform aerobic hydrogen oxidation in marine environments.

### Distribution of hydrogenases in metagenomic databases

Single bacterial strains allow a detailed analysis of part of the genomes that occur in the specific environment they have been isolated from. However, isolated strains provide only a glimpse on the genetic diversity that might be present in the habitat from which they originate, given that most microbial strains are unculturable [52]–[54]. Therefore, we searched the global ocean sampling database (GOS)[32]–[34] with the same representative hydrogenases as given in Table 3 and the representatives of the small hydrogenase subunits (Fig. S5 to S7, supporting information).

This database contains millions of sequence reads that have been obtained mostly from biological samples with a particle size of 0.2 to 0.8 µm. Due to this size fractionation, the major proportion of the sequences belongs to *Pelagibacter ubique* and the *Prochlorococcus*/*Synechococcus* group of cyanobacteria [33]. Since the large number of sequences in the Sargasso Sea metagenome belonging to the *Shewanellaceae* and the *Burkholderiaceae* was discussed to be a contamination [55] Station 11 was not included in the analysis.

**Figure 2:**
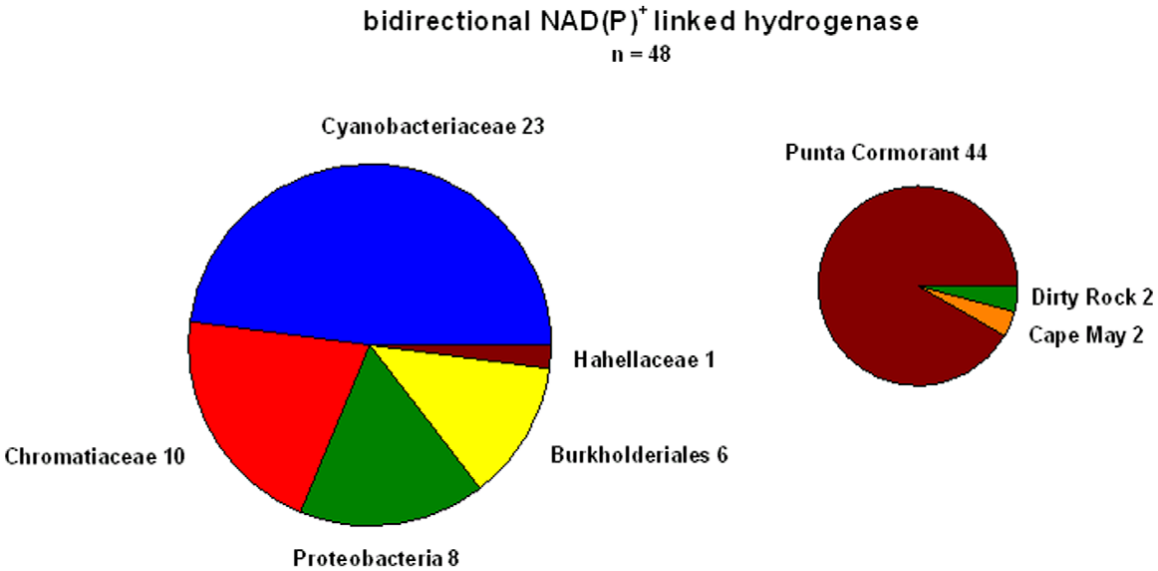
Distribution of bidirectional NAD(P) linked hydrogenases found in the GOS database of the different prokaryotic groups. The *hoxH* sequence of *Synechocystis* sp. PCC 6803 (Table 3) was used for the search and a total of 48 sequences has been found. On the right the number of sequences from the different sampling stations is shown.

We could not detect any cyanobacterial bidirectional hydrogenase in the samples taken from the open ocean. All the cyanobacterial HoxH sequences that could be found in the database are from a single sample taken at Punta Comorant, a hypersaline pond with low oxygen levels [56] on the Galapagos Islands (Fig. 2). These sequences were most similar to the available bidirectional hydrogenases of *Synechococcus* strains (Fig. S8, supporting information). Thus, the GOS sampling and sequencing effort should have been able to capture any HoxH sequence present in the *Prochlorococcus*/*Synechococcus* group. Although it has to be taken into account that environmental sequencing does not capture 100% of the present DNA sequences it seems highly probable that this cyanobacterial hydrogenase is absent in these strains in these environments as already deduced from the whole genomes (Table 2, Table S1, supporting information).

These findings are also corroborated when looking at the *hoxH* sequences of the *Burkholdericeae.* Although these bacteria make up a major fraction of all the oceanic metagenome sequences, there are only representatives from Punta Cormorant with this hydrogenase (Fig. 2), whereas no sequences of this group have been retrieved from the open ocean. Altogether 48 *hoxH* sequences could be found but apart from three coastal stations (Mangrove on Isabella Island, Cape May and Dirty Rock), which accounted for 4 sequences all of the other 44 were exclusively from Punta Cormorant. This confirms the presence of *hoxH* in shallow coastal environments and ponds in a variety of different bacterial groups.

**Figure 3:**
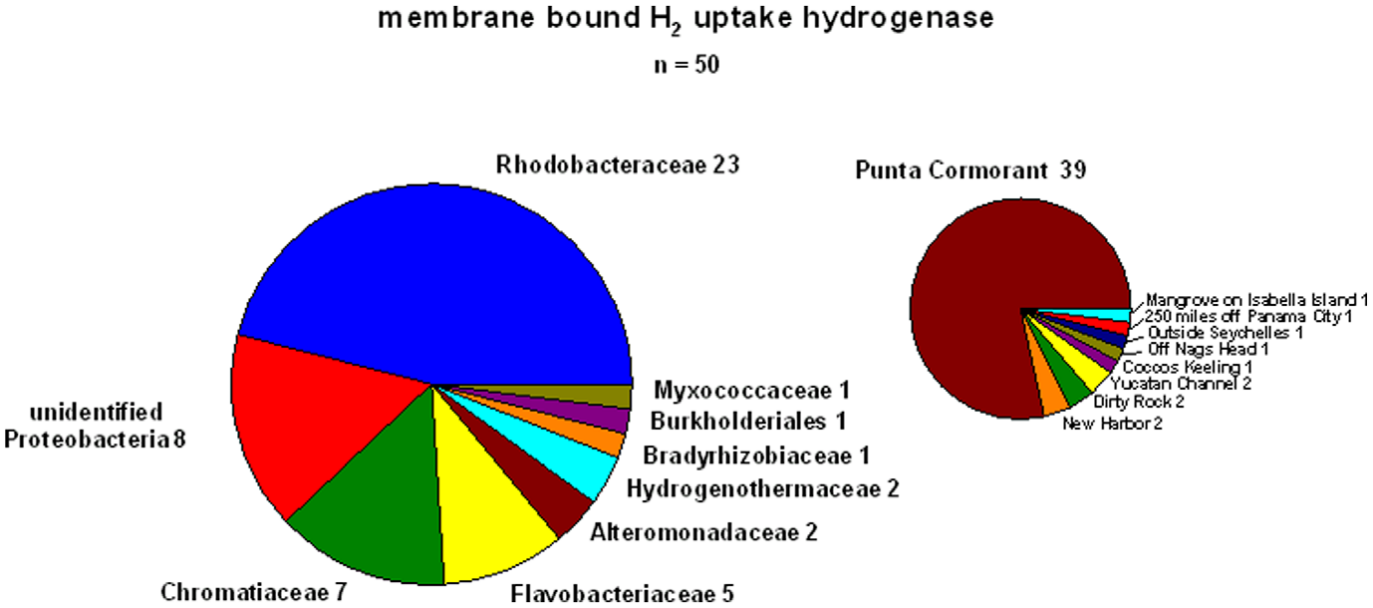
Distribution of membrane-bound hydrogenases found in the GOS database of the different prokaryotic groups. The *hupL* sequence of *Desulfovibrio vulgaris* (Table 3) was used for the search and a total of 51 sequences has been retrieved. On the right the number of sequences from the different sampling stations is shown.

The largest group of sequences in the metagenome database were those of the membrane-bound NiFe-hydrogenases. Again most of the 51 sequences were found at Punta Cormorant, although 11 sequences were detected in the datasets of coastal stations (New Harbor, Dirty Rock, Yucatan Channel, Nags Head, a Mangrove on Isabella Island) and two were found in the open ocean (outside Seychelles and 250 miles of Panama) (Fig. 3).

**Figure 4:**
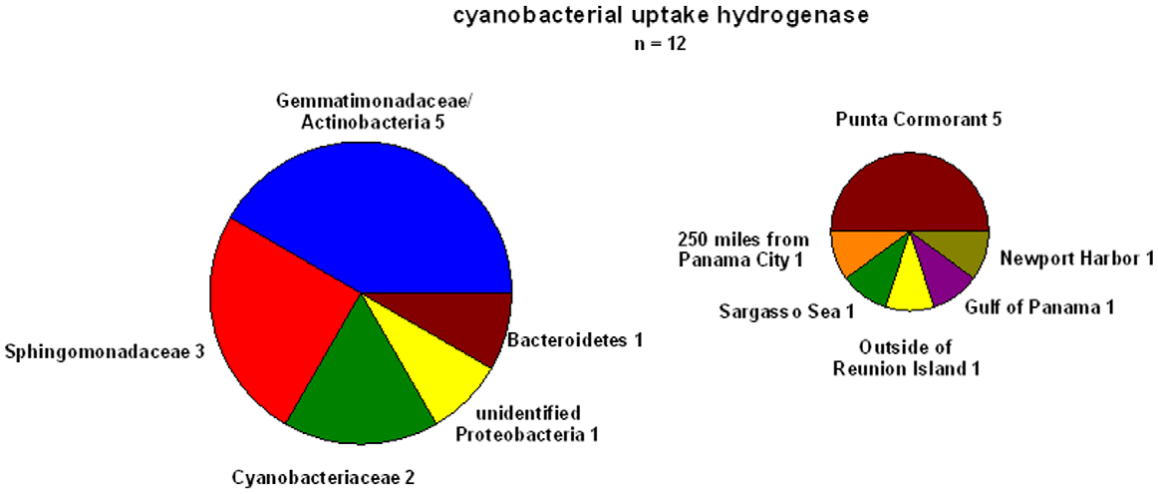
Distribution of cyanobacterial-like uptake hydrogenases found in the GOS database of the different prokaryotic groups. The *hupL* sequence of *Nostoc* sp. PCC 7120 (Table 3) was used for the search and a total of 35 sequences has been retrieved. On the right the number of sequences from the different sampling stations is shown.

Cyanobacterial-like uptake hydrogenases could also be found in the metagenomic dataset (Fig. 4). Because of the size fractionation (0.2–0.8 µm) most of the larger diazotrophic cyanobacteria have been excluded from this analysis. Therefore, although many of the samples have been taken in regions known to be inhabited by this cyanobacterial group only two sequences could be retrieved from the whole dataset. A total of 35 sequences could be found. Most of these sequences originate from coastal sites (28) but four sequences are from the open ocean (Sargasso Sea, Reunion Island and 250 miles off Panama City).

Searches for the small hydrogenase subunit genes retrieved 23 sequences of the bidirectional NAD(P)^+^-linked hydrogenases, 37 of the membrane bound H_2_ uptake hydrogenases and 18 of the cyanobacterial-like uptake hydrogenases. In all these cases the numbers are close to the expected number when comparing the gene sizes of the respective large and small hydrogenase genes (Fig. S5 to S7, supporting information).

**Figure 5:**
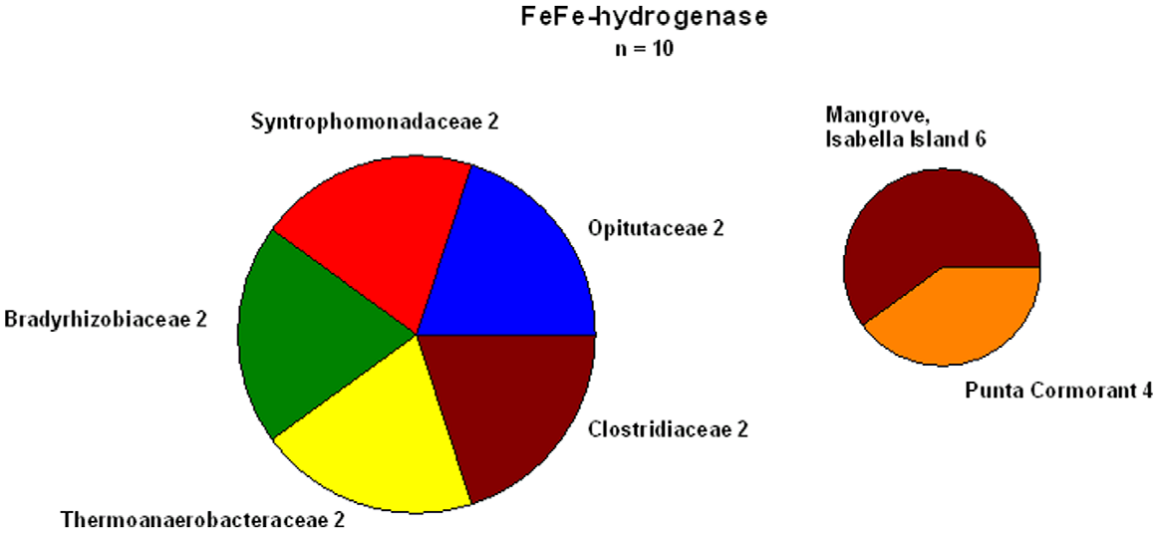
Distribution of FeFe-hydrogenases found in the GOS database of the different prokaryotic groups. The *hydA* sequence of *Clostridium pasteurianum* (Table 3) was used for the search and a total of 10 sequences have been found. On the right the number of sequences from the different sampling stations is shown.

Sequences of the oxygen sensitive FeFe-hydrogenases retrieved from the GOS database were from a Mangrove (Isabella Island) and the hypersaline pond at Punta Cormorant. In all other samples no FeFe-hydrogenase was found (Fig. 5) and none of the archaebacterial hydrogenases were found in the metagenome sequences.

Recently large amounts of metatranscriptomics data became available (e.g. [57]). A search of the respective dataset revealed the presence of three transcripts of membrane-bound H_2_-uptake hydrogenases. One transcript was most similar to a cyanobacterial uptake hydrogenase, one to the *Flavobacteriaceae* and one to the *Bradyrhizobiaceae*. In this dataset only samples from the open ocean are available.

### Detection of sequences of the bidirectional NAD(P)-linked NiFe-hydrogenase in the North Atlantic, Mediterranean Sea, North Sea, Baltic Sea, and two freshwater lakes

Although all NiFe-hydrogenases share two characteristic motifs with altogether four cysteins at the N- and C-terminus for the binding of the NiFe active site, it is impossible to design degenerated primers that bind to the genes of all different classes of these enzymes. Therefore, we limited our effort to a single class and constructed degenerated primers specific for the bidirectional NAD(P)-linked hydrogenases of cyanobacteria, the *Chloroflexaceae* and some proteobacteria. In cyanobacteria this enzyme is known as the bidirectional hydrogenase. It is closely related to the soluble hydrogenase of *Ralstonia eutropha* and the respiratory complex I [58], [59].

**Figure 6:**
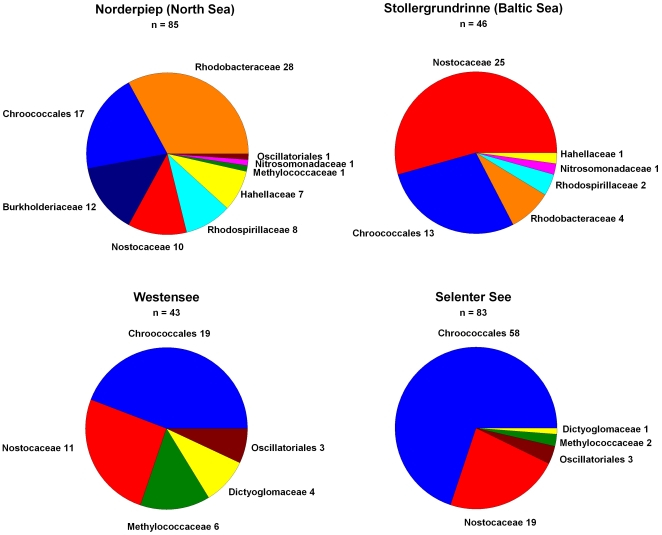
Distribution of bidirectional NAD(P) linked hydrogenases in samples taken from Norderpiep (North Sea), Stollergrundrinne (Baltic Sea) and the freshwater lakes Westensee and Selenter See.

We collected surface water from Stollergrundrinne outside the Kielfjord (Baltic Sea), in the Norderpiep west of Büsum (North Sea) and two freshwater lakes in northern Germany, Westensee and Selenter See. These samples were sequentially filtered on 10 µm and 0.2 µm filters and DNA isolated from the retained material. In samples from all these locations we could detect *hoxH*. In Fig. 6 the distribution of sequences on the different bacterial groups is shown for the different stations.

From the Baltic Sea as well as the fresh water lakes we could amplify a large number of cyanobacterial *hoxH* that are most similar to the *Chroococcales* (most closely related to *Cyanothece*, *Microcystis and Synechocystis*) or the filamentous, heterocystous *Nostocaceae*. In the North Sea the α–proteobacterial group *Rhodobacteraceae* made up the same proportion as all the cyanobacterial sequences taken together. From the freshwater mesotrophic lakes Westensee and Selenter See we could only amplify cyanobacterial *hoxH* (*Chlorococcales*, *Nostocaceae* and *Oscillatoriales*) and in each case some sequences of methylotrophic bacteria and *Dictyoglomaceae*.

In contrast to this, all attempts to amplify sequences of the bidirectional NAD(P)-linked hydrogenases from the samples taken in the North Atlantic off the west African coast and the Ionian Sea (Mediterranean Sea) were negative. This corroborates that the open ocean and marine oligotrophic waters are devoid of this hydrogenase type.

## Discussion

Any conclusion concerning the activity of a gene from its environmental distribution is hampered by the fact that it is not necessarily expressed in a specific environment. Genomes might have genes in store that are not necessary to survive under the present-day conditions, but can be used to invade other niches or to prepare the organism for a drastic change. In the case of the distribution of hydrogenases found in this work, this scenario seems highly unlikely. For several reasons described in detail below, we think that biological hydrogen production and consumption, as depicted in Fig. 6, might be common in a large number of marine and freshwater habitats.

All strains from the open ocean were free of the bidirectional NAD(P) linked hydrogenase. Neither the cyanobacterial genomes nor all of the heterotrophic bacteria (Table 2 and Table S1, supporting information) or the metagenomic sequences harbor this hydrogenase. In addition, our efforts to amplify these hydrogenase genes from the North Atlantic or the Mediterranean Sea were unsuccessful. Since the diazotrophic cyanobacterial strains and the heterotrophic bacteria from the open ocean have other types of hydrogenases, there is no selection pressure against these enzymes per se. However, there is a clear bias of the bidirectional type to environments such as coastal marine waters, ponds, freshwater lakes and microbial mats (Table 2, Fig. 2, Fig. 6 and Table S1, supporting information), where cyanobacteria and heterotrophic bacteria might encounter micro-oxic or anaerobic conditions. In cyanobacteria this type of enzyme was shown to be activated under anaerobiosis and to be responsible for fermentative hydrogen production [60]. This is corroborated by the distribution of the PFOR gene, *nifJ*, in the same cyanobacteria (Table 2).

Starting from anaerobiosis, the bidirectional hydrogenase is known to be used as an electron valve, when cells switch from fermentation to photosynthesis [61]–[64]. These findings might explain the high hydrogen concentration found in the morning hours in a eutrophic lake that coincided with the phytoplankton maximum [13]. Oxygen depletion due to high respiratory activity during the night could have activated the hydrogenase in this zone and elicited a fermentative hydrogen production in the dark that continued at dawn until the next morning when photosynthesis resumed, thus causing supersaturating H_2_ concentrations. A similar diel variation of hydrogen concentrations has also been described for cyanobacterial mats (see e.g. [65]).

In both cases, hydrogen production is certainly not confined to the resident cyanobacteria but can also result from the activity of algae and other heterotrophic bacteria living in the same community.

The large number of genomes of marine bacteria from surface seawaters containing the membrane-bound H_2_-uptake hydrogenase is remarkable. A search of the current marine metatranscriptomics data [57] revealed the expression of these hydrogenases in cyanobacteria as well as other bacteria in the open ocean.

The membrane-bound hydrogenase gene clusters found in the *Rhodobacteraceae* (Fig. S1 supporting information) include all the accessory genes that are known from the membrane-bound hydrogenase of *R. eutropha*. One of the four hydrogenases of *N. caesariensis* and the hydrogenases of the *Roseovarius* strains are closely related to this hydrogenase as revealed by phylogenetic analysis (Fig. S1 and S4, supporting information). This type of enzyme is known to be oxygen insensitive and was shown to be active at ambient oxygen concentrations [66], [67]. Electrochemical investigations of this hydrogenase found measurable hydrogen uptake down to levels of 1 to 10 nM [67], which is well in the range of H_2_ concentrations in surface waters. One of these strains (*Roseovarius* sp. HTCC 2601) was isolated from the Sargasso Sea, but all of the others were from coastal areas. In these regions, this α-proteobacterial subclass makes up as much as 24% of the bacterioplankton [68] and therefore, their hydrogenases might be widespread in these environments.

Mycobacteria, known to colonize aquatic ecosystems, take up hydrogen in the same concentration range under aerobic conditions [69], supporting the notion that hydrogen consumption in these environments is a common microbial feature. Even though the supersaturating concentrations found in surface waters are below the threshold necessary to support growth exclusively on H_2_, hydrogen uptake could add to the ability to survive in a variety of these habitats. Similar suggestions have already been made for hydrogen uptake for long-term survival of bacteria [70] and for the ability to oxidize carbon monoxide in the coastal ocean [71], [72]. These suggestions coincide with the aerobic hydrogen uptake demonstrated for particle sizes between 0.2 and 5 µm in coastal waters [11]. This trait is especially important for litho- and heterotrophic bacteria that have to capitalize on as much of the available energy supply as possible, but can be disregarded by photoautotrophs like cyanobacteria.

Bacterial activity was found to be capable of depleting oxygen in marine organic aggregates. In particles as small as 1.5 mm, anoxic conditions emerged. In the same aggregates no methanogenic or sulfate-reducing bacteria could be detected [73]. Our results suggest that these anaerobic microniches might be specifically occupied by bacteria of the *Vibrionaceae* (Fig. S2, supporting information). Since their membrane-bound H_2_-evolving NiFe-hydrogenases are encoded in conjunction with subunits of the formate dehydrogenase it seems highly likely that it performs the formate:hydrogen lyase reaction. This reaction is well known from *E. coli*, where it detoxifies formate produced during fermentation, evolves hydrogen and might be involved in an additional energy-generating step [24]. The membrane-bound hydrogen uptake hydrogenase encoded in the same genomes would allow hydrogen cycling and might be used for additional net transport of protons across the cell membrane [49].

The *Altermonadaceae* are widespread in marine waters. Two different ecotypes have been sequenced, one is predominant in surface waters whereas the other is known from the deep Mediterranean Sea. The deep ecotype was originally found to harbor the genes of the membrane-bound H_2_ uptake hydrogenase but our analysis and that of others [74] also found the same sequences at the surface of the Sargasso Sea. It was speculated that these two strains are separated by either being associated with small aggregates (surface type) or large aggregates (deep ecotype)[75]. This might be further support for the use of hydrogenases in transiently anoxic microniches in the ocean.

The diel variation of the H_2_ concentration in marine surface waters [8], [9] that parallels solar radiation is still awaiting conclusive explanation. Nitrogen fixation is a major source of hydrogen in terrestrial ecosystems [4]. In-situ measurements of the diazotrophic cyanobacterium *T. thiebautii* suggest that it is a negligible source of hydrogen in the Sargasso Sea [15]. Therefore, nitrogen fixation by filamentous cyanobacteria is an insignificant source of H_2_ in aquatic ecosystems. Interestingly, a unicellular marine diazotrophic cyanobacterium has been shown to be devoid of the uptake hydrogenase [37] and to produce hydrogen while fixing nitrogen [76]. In general unicellular cyanobacteria perform a temporal separation of the oxygen sensitive energy consuming nitrogen fixation process and oxygenic energy generating photosynthesis between night and day, but some strains also fix nitrogen during the light phase [76], [77]. Unicellular strains are known to provide a considerable part of fixed nitrogen in marine waters [78], [79] and might therefore be responsible for part of the evolved H_2_. The newly discovered unicellular cyanobacteria without photosystem II [40], [80] harbor the genes of the cyanobacterial uptake hydrogenase (Table 2), which is most similar to those of the *Cyanothece* group (Fig. S4, supporting information) as expected. Therefore, these strains should be able to recycle the H_2_ evolved by the nitrogenase.

The distribution of cyanobacterial nitrogen fixers in the ocean and their seasonal abundance are poorly characterized although qPCR data has shown that all groups are widely distributed [81], [82]. One investigation suggests that their distribution is patchy and their rate of nitrogen fixation highly variable [79] and might therefore result in hydrogen evolution in some parts and very low or no evolution in other parts.

Although unicellular nitrogen-fixing cyanobacteria might be responsible for hydrogen evolution in some regions, part of the H_2_ produced during the day might be of photochemical origin, such as dissociation of organic matter by UV light [17].

Coastal waters are rich in hydrogenase sequences, as suggested by our analysis of complete genomes (Table 2, 4, Table S1, supporting information), and the number of sequences we could amplify of a single class of NiFe-hydrogenases from the North Sea and the Baltic Sea (Fig. 5). The apparent scarcity of sequences from coastal samples in the GOS database can be explained by the filtration procedure. Since mainly particle sizes between 0.2 and 0.8 µm have been used for DNA isolation many of the coastal bacteria and particle associated bacteria have been excluded from the analysis. We hypothezise that the membrane-bound H_2_ evolving hydrogenase in the genomes of the *Vibrionaceae* might be used as indicator for bacteria that colonize the inner parts of organic aggregates and thus, have not been sequenced yet in the GOS database.

Our analysis shows that the genetic repertoire of bacteria from surface waters of different environments enables them to produce hydrogen either by their nitrogenase, by hydrogenases linked to fermentative pathways (such as the bidirectional NAD(P) linked hydrogenase), or the membrane-bound H_2_-evolving hydrogenase. A number of bacteria could oxidize hydrogen as an energy source probably down to the lower nM range and might be responsible for biological hydrogen consumption in freshwater and marine systems.

This study intends to deliver a first key to the elucidation of the underlying biological processes of hydrogen turnover in aquatic ecosystems. Whether a specific body of water is a hydrogen sink or source will depend on a number of factors such as primary production, nitrogen fixation, the concentration of photodegradable organic compounds and organic particles, and the availability of electron acceptors. This is the first evidence that microorganisms can be an integral part of hydrogen turnover in marine waters, but much more remains to be learned. This is especially true when considering oxygen minimum zones [83] that have not been investigated for the presence of hydrogen or hydrogenases until now.

## Materials and Methods

### Sample collection

Samples were collected from the surface. In the North Sea water was collected in the Norderpiep (54°13′N/8°27′E), in the Baltic Sea it was collected in the Stollergundrinne (54°29′N/10°13′E) and from the freshwater lakes Selenter See (54°18′25N/10°28′53E) and Westensee (54°17′53N/9°57′09E) at least four times a year from every season. These samples were sequentially filtered on 10 µm and 0.2 µm filters with a peristaltic pump 620 S (Watson-Marlow Bredel).

Samples from the Mediterranean Sea were taken from the Ionian Sea at station 2 (36°41′N/21°39′E), station 3 (36°50′N/21°31′E), station 5.2 (36°37′N/21°17′E) and station 6 (36°42′N/21°04′E). In this case 5 l water from a depth of 5 m was filtered on 5 µm and then on 0.2 µm.

The samples from the North Atlantic were taken during the Poseidon 284 cruise at 18°N/30°W, 25°N/30°W and 29°/30°W in April 2002.

For DNA isolation the UltraClean™ Soil DNA Kit (Mo Bio, Carlsbad CA, USA) was used.

### DNA amplification and sequence analysis

Sequences of the bidirectonal NAD(P)-linked hydrogenase were amplified with the primers HoxH-f GTATYTGYGGYATTTGTCCTGT and HoxH-r GGCATTTGTCCTRCTGYATGTGT were used. Prior to 40 cycles of the program the DNA was denatured for 5 min at 95°C. The temperature program was as follows: 30 sec at 95°C, 40 sec at 50°C, 2 min at 72°C. In a final step the temperature was kept at 72°C for 10 min. The reaction contained 0.5 µM of the two primers, 0.2 mM of dNTPs, 2.5 mM MgCl_2_, 0.025 U/µl Taq polymerase (MBI Fermentas, St. Leon-Roth, Germany) and 10x buffer as recommended by the manufacturer in a total volume of 50 µl. Of each sample different amounts of DNA between 2 and 100 ng were tested as template. If no PCR product was detected DNA concentrations were increased at least 10 times. Positive controls were run in parallel to prove the efficiency of the PCR. The approximate size of the product is around 1190 bp and covers close to 84% of the *hoxH* gene.

The resulting PCR products were ligated into the pCRII-topo (Invitrogen), sequenced with the Big-Dye Kit, and applied on a 96 capillary sequencer (3730 DNA Analyzer, Applied Biosystems).

If possible contigs were assembled from the obtained sequence data and the respective sequences deposited in the genebank (Accession numbers GQ454414 to GQ454443 and GU238237 to GU238258) including two additional cyanobacterial *hoxH* sequences of *Aphanothece halophytica* and *Mastigocladus laminosus* SAG 4.84 (Accession numbers GQ454444 and GQ454445).

### Database searches

The genebank, cyanobase, and the GOS database were searched for hydrogenase specific sequences by using the hydrogenase sequences given in Table 3. Retrieved sequences were either run against the genebank by using the BLAST algorithm [38] to deduce the closest homolog or searched for the signature sequences as given by Vignais and Billoud [19] to unambiguously classify the respective hydrogenase. In case of the GOS database, the sequences found were aligned, and, if possible, larger contigs were formed from the same sampling station and used for all further analysis.

### Phylogenetic analysis

In the case of critical candidates or unclear phylogenetic affiliation phylogenetic trees were used. Sequence alignments were made with ClustalW [84]. After manual optimization and removal of gaps from the alignments, parsimony, maximum likelihood, and distances were calculated with the 3.63 release of the PHYLIP package [85], using the Jones-Taylor-Thornton matrix and the algorithm of Fitch and Margoliash [86]. Maximum parsimony and distances were calculated for 1000 bootstraps and maximum likelihood for 100 bootstraps. The Unix-cluster at the computer center of the University of Kiel was used for most of the calculations. The resulting trees are given in Fig. S3 to S5 (supporting information).

## Supporting Information

Table S1 Complete list of all marine bacteria searched for hydrogenase genes
(0.05 MB XLS)

Figure S1 Structure of the gene cluster of the membrane bound hydrogen uptake NiFe-hydrogenase of marine *Rhodobacteraceae* and the delta-proteobacterium *Neptuniibacter caesariensis*. The structural genes of the hydrogenase (hupS, hupL and hupZ the membrane bound cytochtrome) are shown in blue. Red genes (hoxAJBC) are involved in the regulation of the hydrogenase. HoxJ encodes a histidine kinase that is known to interact with a hydrogen sensor encoded by hoxB and hoxC and regulates the activity of the response regulator encoded by hoxA. HupK might encode a protein necessary to express an oxygen-tolerant hydrogenase. Accessory genes known to be necessary for this type of membrane hydrogenase are shown in grey, whereas grey patterned genes are general accessory genes for all NiFe-hydrogenases. Genes depicted in green are putative proteases that cleave the C-terminus of the hydrogenase. HypX of *Ralstonia eutropha* is known to render its soluble hydrogenase oxygen tolerant.
(0.06 MB DOC)

Figure S2 Structure of three hydrogenase gene clusters of *Vibrioanceae* isolated from marine environments that are most similar to the energy converting H2-evolving NiFe-hydrogenases. The color code is the same as in Figure S1. Genes shown in plaid are part of the fromate dehydrogenase. FhlA is the transcriptional activator of the formate-hydrogen lyase. Those in black and grey-blue are additional subunits of the whole complex.
(0.05 MB DOC)

Figure S3 Phylogenetic tree of HypX. Representatives of enoyl-CoA hydratase/crotonase have been used as outgroup. The abbreviations and the respective accession numbers are as follows: Aaeoli, Aquifex aeolicus VF5 NP_213788; Aehrli, Alkalilimnicola ehrlichei MLHE-1 YP_742845; Amarin, Acaryochloris marina MBIC11017 YP_001520946; BjapUSDA, Bradyrhizobium japonicum USDA 110 NP_773566; Cviola, Chromobacterium violaceum ATCC 12472 NP_903812; Daroma, Dechloromonas aromatica RCB YP_287160; Frankia Cc Frankia sp. CcI3 YP_482743; Frankia EA Frankia sp. EAN1pec YP_001505433; MmagAMB, Magnetospirillum magneticum AMB-1 YP_420998; MmagMS-1, Magnetospirillum magnetotacticum MS-1 ZP_00055441; Mmarina Microscilla marina ATCC 23134 ZP_01691397; Mpetro, Methylibium petroleiphilum PM1 YP_001021998; Ncaesar, Neptuniibacter caesariensis ZP_01166042; Nitrati, Nitratiruptor sp. SB155-2 YP_001356445; Pedobac Pedobacter sp. BAL39 ZP_01883353; Pnapht, Polaromonas naphthalenivorans CJ2 YP_982187, PfluPF-5, Pseudomonas fluorescens Pf-5 YP_260772; Pfluore, Pseudomonas fluorescens PfO-1 YP_348856; Reutro, Ralstonia eutropha H16 NP_942660; Rferri, Rhodoferax ferrireducens T118 YP_525330; Rmetalli, Ralstonia metallidurans CH34 YP_583693; Saverm, Streptomyces avermitilis MA-4680 NP_828541; Savermi Streptomyces avermitilis MA-4680 NP_823962; Scoelic Streptomyces coelicolor A3(2) NP_629596; Sdegra, Saccharophagus degradans 2-40 YP_526001; Smalto Stenotrophomonas maltophilia R551-3 YP_002027502; Ssedimi, Shewanella sediminis HAW-EB3 YP_001475080; Sulfuro, Sulfurovum sp. NBC37-1 YP_001358952; Xcamp Xanthomonas campestris pv. vesicatoria str. 85-10 YP_363011
(0.50 MB DOC)

Figure S4 Phylogenetic tree of HupL sequences. Representatives of the 49 kDa subunit of the complex I have been used as outgroup. The used abbreviations and their respective accession numbers are as follows: Abac345 Candidatus Koribacter versatilis Ellin345 YP_593314; Abut4018 Arcobacter butzleri RM4018 YP_001490358; Afer53993 Acidithiobacillus ferrooxidans ATCC 53993 YP_002219307; Ahyd7966 Aeromonas hydrophila subsp. hydrophila ATCC 7966 YP_857036; AmacDE Alteromonas macleodii ‘Deep ecotype’ YP_002124659; Aple4074 Actinobacillus pleuropneumoniae serovar 1 str. 4074 ZP_00134404; AsalA449 Aeromonas salmonicida subsp. salmonicida A449 YP_001141617; Asiam Anabaena siamensis TISTR 8012 AAN65266; Avar Anabaena variabilis ATCC 29413 YP_325087; Bac Ellin bacterium Ellin514 ZP_03626632; BBTAi1-2 Bradyrhizobium sp. BTAi1 YP_001220511; BBTAi1-3 Bradyrhizobium sp. BTAi1 YP_001236652; Bjap110 Bradyrhizobium japonicum USDA 110 NP_773581; Bphy815 Burkholderia phymatum STM815 YP_001863308; C.fer13031 Chlorobium ferrooxidans DSM 13031 ZP_01386726; C511412 Cyanothece sp. ATCC 51142 YP_001802481; C7424 Cyanothece sp. PCC 7424 YP_002377118; C7822 Cyanothece sp. PCC 7822 ZP_03153783; C8802 Cyanothece sp. PCC 8802 ZP_03142797; Cagg Chloroflexus aggregans DSM 9485 YP_002461848; Caur10-fl Chloroflexus aurantiacus J-10-fl YP_001636362; CCY0110 Cyanothece sp. CCY 0110 ZP_01728928; Chyd Carboxydothermus hydrogenoformans Z-2901 YP_360377; Cjej1221 Campylobacter jejuni RM1221 YP_179388; Ckos895 Citrobacter koseri ATCC BAA-895 YP_001455880; Clim245 Chlorobium limicola DSM 245 YP_001942914; CmedTB-2 Caminibacter mediatlanticus TB-2 ZP_01871651; Cpha Chlorobium phaeobacteroides DSM 266 YP_911445; CtepTLS Chlorobium tepidum TLS NP_661672; Cwat8501 Crocosphaera watsonii WH 8501 ZP_00519188; Dbac Desulfomicrobium baculatum 1CC1_L; DBAV1 Dehalococcoides sp. BAV1 YP_001213724; Deth Dehalococcoides ethenogenes 195 YP_180861; DvulDP4 Desulfovibrio vulgaris DP4 YP_966691; Ecar1043 Pectobacterium atrosepticum SCRI1043 YP_049334; EcolK12 Escherichia coli str. K-12 substr. MG1655 NP_415492; EcolNuoD Escherichia coli CAA48363; FACN14a Frankia alni ACN14a YP_712616; FACN14a-2 Frankia alni ACN14a YP_712064; Fbac Flavobacteria bacterium MS024-2A ZP_03702421; FCci3 Frankia sp. CcI3 YP_481046; FEAN Frankia sp. EAN1pec YP_001506830; FEAN2 Frankia sp. EAN1pec YP_001507712; Gaur Gemmatimonas aurantiaca T-27 YP_002759924; Gloeo Gloeothece sp. PCC 6909 AAP04005; GlovSZ Geobacter lovleyi SZ YP_001952291; GlovSZ-2 Geobacter lovleyi SZ YP_001950403; HpylJ99 Helicobacter pylori J99 NP_223293; L8106 Lyngbya sp. PCC 8106 ZP_01619041; Laes Lyngbya aestuarii ABD34838; Lint Lawsonia intracellularis PHE/MN1-00 YP_594816; Lmaj Lyngbya majuscula CCAP 1446/4 AAO66476; Mavi Mycobacterium avium 104 YP_881873; MJLS Mycobacterium sp. JLS YP_00107040; Mkan Mycobacterium kansasii ATCC 12478 ZP_04750138; Mmag-1-3 Magnetospirillum magneticum AMB-1 YP_421305; MmagMS-1 Magnetospirillum magnetotacticum MS-1 ZP_00052632; Mmar Mycobacterium marinum M YP_001850173; MMCS Mycobacterium sp. MCS YP_639307; Msil Methylocella silvestris BL2 YP_002364007; Msme Mycobacterium smegmatis str. MC2 155 YP_887053; N7120 Nostoc sp. PCC 7120 NP_484720; N7422 Nostoc sp. PCC 7422 BAE46791; Nazo ‘Nostoc azollae’ 0708 ZP_03768004; Neptuni2 Neptuniibacter caesariensis ZP_01167270; Neptuni 1 Neptuniibacter caesariensis ZP_01166595; Npun Nostoc punctiforme PCC 73102 AAC16277; Nspu Nodularia spumigena CCY 9414 ZP_01628406; Paes Prosthecochloris aestuarii DSM 271 YP_002015547; Pars Pyrobaculum arsenaticum DSM 13514 YP_001153513; Pdis8503 Parabacteroides distasonis ATCC 8503 YP_001303173; Photob34 Photobacterium sp. SKA34 ZP_01160131; Pisl Pyrobaculum islandicum DSM 4184 YP_929722; Plut Pelodictyon luteolum DSM 273 YP_375349; PMED4NdH Prochlorococcus marinus subsp. pastoris str. CCMP1986 NP_892293; Ppha Pelodictyon phaeoclathratiforme BU-1 YP_002018704; Rcap Rhodobacter capsulatus AAA69668; Rcas Roseiflexus castenholzii DSM 13941 YP_001433219; Rcas2 Roseiflexus castenholzii DSM 13941 YP_001433862; ReryPR4 Rhodococcus erythropolis PR4 YP_002766098; RerySK121 Rhodococcus erythropolis SK121 ZP_04384689; Reut Ralstonia eutropha H16 NP_942704; ReutC Ralstonia eutropha H16 NP_942663; ReutG Ralstonia eutropha H16 AAA16462; Rgel Methylibium petroleiphilum PM1 YP_001022015; RHTCC2501 Robiginitalea biformata HTCC2501 ZP_01119574; Rhtcc2601 Roseovarius sp. HTCC2601 ZP_01443057; RHTCC2601-Sens Roseovarius sp. HTCC2601 ZP_01443054; Rjos Rhodococcus jostii RHA1 YP_704548; Ropa Rhodococcus opacus B4 YP_002781742; Rpal009 Rhodopseudomonas palustris CGA009 NP_946314; RpalA53 Rhodopseudomonas palustris BisA53 YP_780164; RpalB5 Rhodopseudomonas palustris BisB5 YP_568300; RRS-1 Roseiflexus sp. RS-1 YP_001276649; Rrub Rhodospirillum rubrum ATCC 11170 YP_426250; Rsph17029 Rhodobacter sphaeroides ATCC 17029 YP_001044019; Rsph2.4.1 Rhodobacter sphaeroides 2.4.1 YP_353568; Rtm1035 Roseovarius sp. TM1035 ZP_01881109; Sag12614 Stappia aggregata IAM 12614 ZP_01550392; Sag12614-2 Stappia aggregata IAM 12614 ZP_01550270; Sala2256 Sphingopyxis alaskensis RB2256 YP_611130; Sama Shewanella amazonensis SB2B YP_927554; Save Streptomyces avermitilis MA-4680 NP_828543; SbalOS155 Shewanella baltica OS155 YP_001050263; Sdys197 Shigella dysenteriae Sd197 YP_402612; SentATCC Salmonella enterica subsp. enterica serovar Paratyphi A str. ATCC 9150 YP_152163; SentCT18 Salmonella enterica subsp. enterica serovar Typhi str. CT18 NP_456296; SfumMPOB Syntrophobacter fumaroxidans MPOB YP_847061; Slin Spirosoma linguale DSM 74 ZP_04492490; SoneMR-1 Shewanella oneidensis MR-1 NP_717701; SoneMR-4 Shewanella sp. MR-4 YP_733952; SoneMR-7 Shewanella sp. MR-7 YP_738201; Sros Streptosporangium roseum DSM 43021 ZP_04474993; Sste37 Sagittula stellata E-37 ZP_01748533; Ssvi Streptomyces sviceus ATCC 29083 YP_002204206; Susi Solibacter usitatus Ellin6076 YP_827763; Svir Saccharomonospora viridis DSM 43017 ZP_04507584; TcarNor1 Thermosinus carboxydivorans Nor1 ZP_01667576; Tden25259 Thiobacillus denitrificans ATCC 25259 YP_315133; Tden33889 Sulfurimonas denitrificans DSM 1251 YP_393947; Tery Trichodesmium erythraeum IMS101 YP_722943; TM1035-Sens Roseovarius sp. TM1035 ZP_01881113; Tros 5159 Thermomicrobium roseum DSM 5159 YP_002523076; Tros2 Thiocapsa roseopersicina AAA27410; Tros Thiocapsa roseopersicina AAC38282; Ucyn-A Cyanothece sp. CCY 0110 ZP_01728928; VangS14 Vibrio angustum S14 ZP_01234606; Wsuc1740 Wolinella succinogenes DSM 1740 NP_907813; Yent8081 Yersinia enterocolitica subsp. enterocolitica 8081 YP_001007767. The sequence of the marine unicellular group A cyanobacteria has been generated from the available short reads [70].
(0.67 MB DOC)

Figure S5 Distribution of small subunits of the bidirectional NAD(P)+ linked hydrogenase found in the GOS database of the different prokaryotic groups. The small subunit gene, hoxY, of Synechocystis has been used for the search. All genes have been retrieved form Punta Comorant, a hypersaline pond on the Galapagos Islands.
(0.06 MB DOC)

Figure S6 Distribution of small subunits of the membrane bound H2 uptake hydrogenasses found in the GOS database of the different prokaryotic groups. The hupS sequence of Desulfovibrio vulgaris was used for the search. On the right the number of sequences from the different sampling stations is shown.
(0.08 MB DOC)

Figure S7 Distribution of small subunits of the cyanobacterial-like uptake hydrogenase found in the GOS database of the different prokaryotic groups. The small subunit gene, hupS, of Nostoc sp. PCC 7120 has been used for the search.
(0.05 MB DOC)

Figure S8 Phylogenetic tree of HoxH sequences. Representatives of the 49 kDa subunit of the complex I have been used as outgroup. The used abbreviations and their respective accession numbers are as follows: Afla Acetomicrobium flavidum CAA56464; Ahalo Aphanothce halophytica GQ454444; Amar Acaryochloris marina MBIC11017 YP_001521996; Amax Arthrospira maxima FACHBSM AAQ63961; Apla1 Arthrospira platensis FACHB341 AAQ63964; Apla2 Arthrospira platensis FACHBOUQDS6 AAQ63959; Apla3 Arthrospira platensis FACHB439 AAQ63960; Apla4 Arthrospira platensis FACHB791 AAQ91344; Avar Anabaena variabilis ATCC 29413 YP_325153; Bxen Burkholderia xenovorans LB400 YP_555781; Cagg Chloroflexus aggregans DSM 9485 YP_002463784; CaggL Chlorobium chlorochromatii CaD3 YP_378564; Caur Chloroflexus aurantiacus J-10-fl YP_001634807; CCY0110 Cyanothece sp. CCY 0110 ZP_01727423; ClimL Chlorobium limicola DSM 245 YP_001944104; Cnec Ralstonia eutropha H16 NP_942730; CphaL Chlorobium phaeobacteroides DSM 266 YP_912598;CtepL Chlorobium tepidum TLS NP_662771; Daro Dechloromonas aromatica RCB YP_284208; DethV Dehalococcoides ethenogenes 195 YP_181357; Dpsy Desulfotalea psychrophila LSv54 YP_065948; DpsyV Desulfotalea psychrophila LSv54 YP_064749;Ecol Escherichia coli CAA48363; Galp Gloeocapsa alpicola str. CALU 743 AAO85440; Gmet1 Geobacter metallireducens GS-15 YP_384078; Gmet2 Geobacter metallireducens GS-15 YP_386258; GOS1 and GOS2 are the two consenus sequeces retrieved from the GOS database; Gsul1 Geobacter sulfurreducens PCA NP_953465; Gsul2 Geobacter sulfurreducens PCA NP_953763; Lyng Lyngbya majuscula CCAP 1446/4 AAT07678; Magneto Magnetococcus sp. MC-1 YP_864809; Mastigo Mastigocladus laminosus SAG 4.84 GQ454445; Mcap Methylococcus capsulatus str. Bath YP_112653; MferV Methanothermus fervidus Q49179; MjanV Methanocaldococcus jannaschii DSM 2661 NP_248187; Mkan Methanopyrus kandleri AV19 NP_613553; Mmag Magnetospirillum magnetotacticum MS-1 ZP_00053777; MmarV Methanococcus maripaludis S2 NP_987943;MvolV1 Methanococcus voltae Q00404; MvolV2 Methanococcus voltae Q00407;N7120 Nostoc sp. PCC 7120 NP_484809; N7422 Nostoc sp. PCC 7422 BAE46796; Neptuni Oceanospirillum sp. MED92 ZP_01164927; Nitrococcus Nitrococcus mobilis Nb-231 ZP_01126922; Nspu Nodularia spumigena CCY 9414 ZP_01629499; Nspu Nodularia spumigena CCY 9414 ZP_01629499; PaesL Prosthecochloris aestuarii DSM 271 YP_002016588; PfurL1 Pyrococcus furiosus DSM 3638 NP_578623; PfurL2 Pyrococcus furiosus DSM 3638 NP_579061; Phol Prochlorothrix hollandica AAB53705;Plancto Planctomyces maris DSM 8797 ZP_01852867; PMED4 Prochlorococcus marinus subsp. pastoris str. CCMP1986 NP_892293; PphaL Pelodictyon phaeoclathratiforme BU-1 YP_002019299; Rcas Roseiflexus castenholzii DSM 13941 YP_001431482; Rmet Ralstonia metallidurans CH34 YP_583677; Ropa Rhodococcus opacus AAB57892; RRS-1 Roseiflexus sp. RS-1 YP_001277847; S6301 Synechococcus elongatus PCC 6301 YP_172265; S6803 Synechocystis sp. PCC 6803 NP_441259; S6803 Synechocystis sp. PCC 6803 NP_441411;S7002 Synechococcus sp. PCC 7002 YP_001733469; S7942 Synechococcus elongatus PCC 7942 YP_401572; Spla Arthrospira platensis FACHB440 AAQ63963; Ssub Spirulina subsalsa FACHB351 AAQ63962; Susi Solibacter usitatus Ellin6076 YP_826256;Tros Thiocapsa roseopersicina AAP50523; WH5701 Synechococcus sp. WH 5701 ZP_01085930.
(0.13 MB DOC)
