# Supplementary material for: Distribution Analysis of Hydrogenases in Surface Waters of Marine and Freshwater Environments
Source: PLoS One. 2010 Nov 5;5(11):e13846. doi: 10.1371/journal.pone.0013846 (PMC2974642; doi:10.1371/journal.pone.0013846)
Supplement: Figure S1 — Structure of the gene cluster of the membrane bound hydrogen uptake NiFe-hydrogenase of marine Rhodobacteraceae and the delta-proteobacterium Neptuniibacter caesariensis. The structural genes of the hydrogenase (hupS, hupL and hupZ the membrane bound cytochtrome) are shown in blue. Red genes (hoxAJBC) are involved in the regulation of the hydrogenase. HoxJ encodes a histidine kinase that is known to interact with a hydrogen sensor encoded by hoxB and hoxC and regulates the activity of the response regulator encoded by hoxA. HupK might encode a protein necessary to express an oxygen-tolerant hydrogenase. Accessory genes known to be necessary for this type of membrane hydrogenase are shown in grey, whereas grey patterned genes are general accessory genes for all NiFe-hydrogenases. Genes depicted in green are putative proteases that cleave the C-terminus of the hydrogenase. HypX of Ralstonia eutropha is known to render its soluble hydrogenase oxygen tolerant. (0.06 MB DOC) [file pone.0013846.s002.doc]

§

Fig. S1: Structure of the gene cluster of the membrane bound hydrogen uptake NiFe-hydrogenase of marine *Rhodobacteraceae* and the δ-proteobacterium *Neptuniibacter caesariensis*. The structural genes of the hydrogenase (hupS, hupL and hupZ the membrane bound cytochtrome) are shown in blue. Red genes (*hoxAJBC*) are involved in the regulation of the hydrogenase. *HoxJ* encodes a histidine kinase that is known to interact with a hydrogen sensor encoded by *hoxB* and *hoxC* and regulates the activity of the response regulator encoded by *hoxA*. *HupK* might encode a protein necessary to express an oxygen-tolerant hydrogenase. Accessory genes known to be necessary for this type of membrane hydrogenase are shown in grey, whereas grey patterned genes are general accessory genes for all NiFe-hydrogenases. Genes depicted in green are putative proteases that cleave the C-terminus of the hydrogenase. *HypX* of *Ralstonia eutropha* is known to render its soluble hydrogenase oxygen tolerant.
