# Supplementary material for: Distribution Analysis of Hydrogenases in Surface Waters of Marine and Freshwater Environments
Source: PLoS One. 2010 Nov 5;5(11):e13846. doi: 10.1371/journal.pone.0013846 (PMC2974642; doi:10.1371/journal.pone.0013846)
Supplement: Figure S2 — Structure of three hydrogenase gene clusters of Vibrioanceae isolated from marine environments that are most similar to the energy converting H2-evolving NiFe-hydrogenases. The color code is the same as in Figure S1. Genes shown in plaid are part of the fromate dehydrogenase. FhlA is the transcriptional activator of the formate-hydrogen lyase. Those in black and grey-blue are additional subunits of the whole complex. (0.05 MB DOC) [file pone.0013846.s003.doc]

§

Fig. S2 :. Structure of three hydrogenase gene clusters of *Vibrioanceae* isolated from marine environments that are most similar to the energy converting H_2_-evolving NiFe-hydrogenases. The color code is the same as in Figure S1. Genes shown in plaid are part of the fromate dehydrogenase. FhlA is the transcriptional activator of the formate-hydrogen lyase. Those in black and grey-blue are additional subunits of the whole complex.
