# Supplementary material for: Distribution Analysis of Hydrogenases in Surface Waters of Marine and Freshwater Environments
Source: PLoS One. 2010 Nov 5;5(11):e13846. doi: 10.1371/journal.pone.0013846 (PMC2974642; doi:10.1371/journal.pone.0013846)
Supplement: Figure S3 — Phylogenetic tree of HypX. Representatives of enoyl-CoA hydratase/crotonase have been used as outgroup. The abbreviations and the respective accession numbers are as follows: Aaeoli, Aquifex aeolicus VF5 NP_213788; Aehrli, Alkalilimnicola ehrlichei MLHE-1 YP_742845; Amarin, Acaryochloris marina MBIC11017 YP_001520946; BjapUSDA, Bradyrhizobium japonicum USDA 110 NP_773566; Cviola, Chromobacterium violaceum ATCC 12472 NP_903812; Daroma, Dechloromonas aromatica RCB YP_287160; Frankia Cc Frankia sp. CcI3 YP_482743; Frankia EA Frankia sp. EAN1pec YP_001505433; MmagAMB, Magnetospirillum magneticum AMB-1 YP_420998; MmagMS-1, Magnetospirillum magnetotacticum MS-1 ZP_00055441; Mmarina Microscilla marina ATCC 23134 ZP_01691397; Mpetro, Methylibium petroleiphilum PM1 YP_001021998; Ncaesar, Neptuniibacter caesariensis ZP_01166042; Nitrati, Nitratiruptor sp. SB155-2 YP_001356445; Pedobac Pedobacter sp. BAL39 ZP_01883353; Pnapht, Polaromonas naphthalenivorans CJ2 YP_982187, PfluPF-5, Pseudomonas fluorescens Pf-5 YP_260772; Pfluore, Pseudomonas fluorescens PfO-1 YP_348856; Reutro, Ralstonia eutropha H16 NP_942660; Rferri, Rhodoferax ferrireducens T118 YP_525330; Rmetalli, Ralstonia metallidurans CH34 YP_583693; Saverm, Streptomyces avermitilis MA-4680 NP_828541; Savermi Streptomyces avermitilis MA-4680 NP_823962; Scoelic Streptomyces coelicolor A3(2) NP_629596; Sdegra, Saccharophagus degradans 2-40 YP_526001; Smalto Stenotrophomonas maltophilia R551-3 YP_002027502; Ssedimi, Shewanella sediminis HAW-EB3 YP_001475080; Sulfuro, Sulfurovum sp. NBC37-1 YP_001358952; Xcamp Xanthomonas campestris pv. vesicatoria str. 85-10 YP_363011 (0.50 MB DOC) [file pone.0013846.s004.doc]

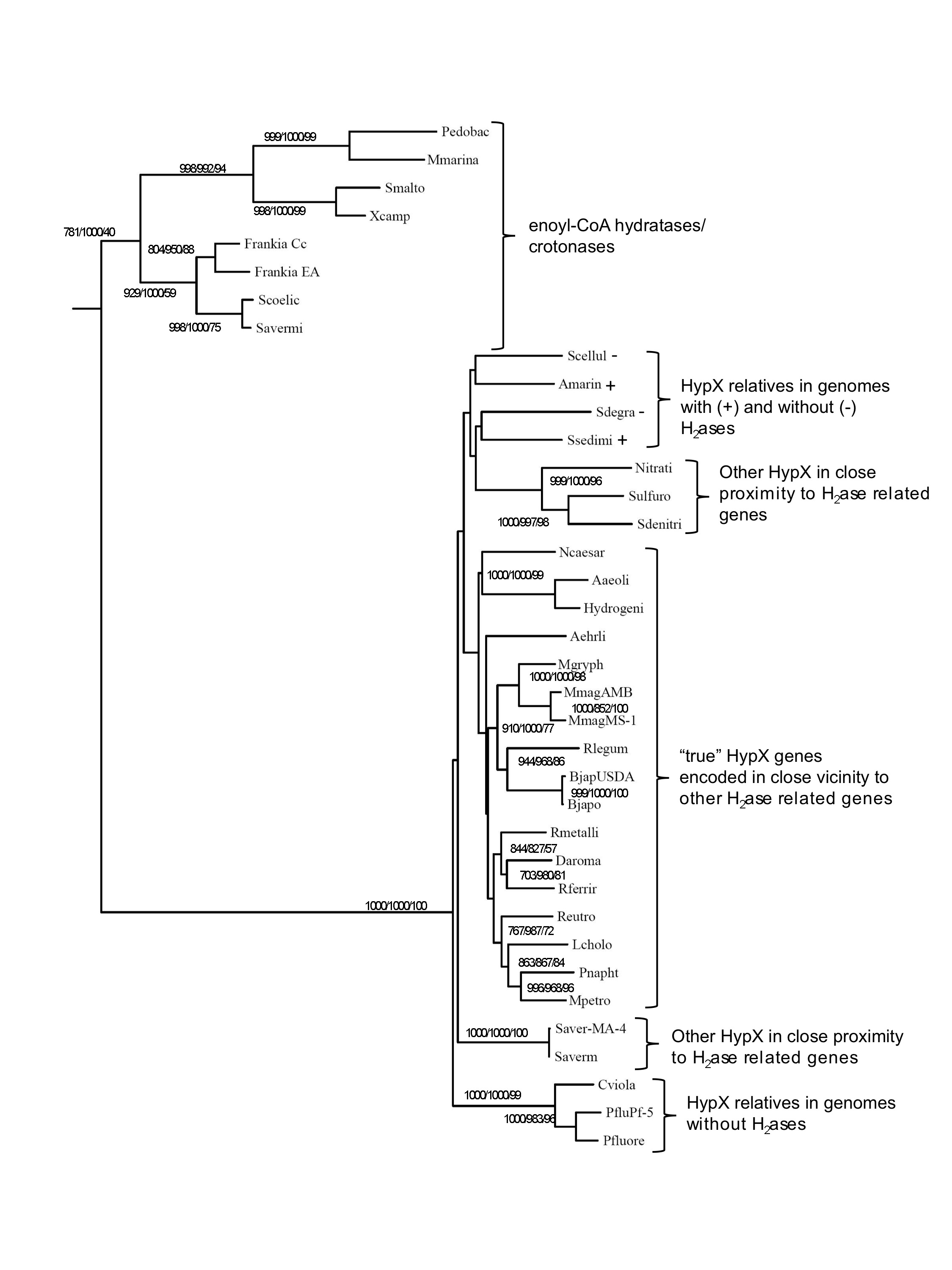


Fig. S3: Phylogenetic tree of HypX. Representatives of enoyl-CoA hydratase/crotonase have been used as outgroup. The abbreviations and the respective accession numbers are as follows: Aaeoli, *Aquifex aeolicus* VF5 NP_213788; Aehrli, *Alkalilimnicola ehrlichei* MLHE-1 YP_742845; Amarin, *Acaryochloris marina* MBIC11017 YP_001520946; BjapUSDA, *Bradyrhizobium japonicum* USDA 110 NP_773566; Cviola, *Chromobacterium violaceum* ATCC 12472 NP_903812; Daroma, *Dechloromonas aromatica* RCB YP_287160; Frankia Cc *Frankia* sp. CcI3 YP_482743; Frankia EA *Frankia* sp. EAN1pec YP_001505433; MmagAMB, *Magnetospirillum magneticum* AMB-1 YP_420998; MmagMS-1, *Magnetospirillum magnetotacticum* MS-1 ZP_00055441; Mmarina *Microscilla marina* ATCC 23134 ZP_01691397; Mpetro, *Methylibium petroleiphilum* PM1 YP_001021998; Ncaesar, *Neptuniibacter caesariensis* ZP_01166042; Nitrati, *Nitratiruptor* sp. SB155-2 YP_001356445; Pedobac Pedobacter sp. BAL39 ZP_01883353; Pnapht, *Polaromonas naphthalenivorans* CJ2 YP_982187, PfluPF-5, *Pseudomonas fluorescens* Pf-5 YP_260772; Pfluore, *Pseudomonas fluorescens* PfO-1 YP_348856; Reutro, *Ralstonia eutropha* H16 NP_942660; Rferri, *Rhodoferax ferrireducens* T118 YP_525330; Rmetalli, *Ralstonia metallidurans* CH34 YP_583693; Saverm, *Streptomyces avermitilis* MA-4680 NP_828541; Savermi *Streptomyces avermitilis* MA-4680 NP_823962; Scoelic *Streptomyces coelicolor* A3(2) NP_629596; Sdegra, *Saccharophagus* *degradans* 2-40 YP_526001; Smalto *Stenotrophomonas maltophilia* R551-3 YP_002027502; Ssedimi, *Shewanella sediminis* HAW-EB3 YP_001475080; Sulfuro, *Sulfurovum* sp. NBC37-1 YP_001358952; Xcamp *Xanthomonas campestris* pv. *vesicatoria* str. 85-10 YP_363011;
