# Supplementary material for: Distribution Analysis of Hydrogenases in Surface Waters of Marine and Freshwater Environments
Source: PLoS One. 2010 Nov 5;5(11):e13846. doi: 10.1371/journal.pone.0013846 (PMC2974642; doi:10.1371/journal.pone.0013846)
Supplement: Figure S4 — Phylogenetic tree of HupL sequences. Representatives of the 49 kDa subunit of the complex I have been used as outgroup. The used abbreviations and their respective accession numbers are as follows: Abac345 Candidatus Koribacter versatilis Ellin345 YP_593314; Abut4018 Arcobacter butzleri RM4018 YP_001490358; Afer53993 Acidithiobacillus ferrooxidans ATCC 53993 YP_002219307; Ahyd7966 Aeromonas hydrophila subsp. hydrophila ATCC 7966 YP_857036; AmacDE Alteromonas macleodii ‘Deep ecotype’ YP_002124659; Aple4074 Actinobacillus pleuropneumoniae serovar 1 str. 4074 ZP_00134404; AsalA449 Aeromonas salmonicida subsp. salmonicida A449 YP_001141617; Asiam Anabaena siamensis TISTR 8012 AAN65266; Avar Anabaena variabilis ATCC 29413 YP_325087; Bac Ellin bacterium Ellin514 ZP_03626632; BBTAi1-2 Bradyrhizobium sp. BTAi1 YP_001220511; BBTAi1-3 Bradyrhizobium sp. BTAi1 YP_001236652; Bjap110 Bradyrhizobium japonicum USDA 110 NP_773581; Bphy815 Burkholderia phymatum STM815 YP_001863308; C.fer13031 Chlorobium ferrooxidans DSM 13031 ZP_01386726; C511412 Cyanothece sp. ATCC 51142 YP_001802481; C7424 Cyanothece sp. PCC 7424 YP_002377118; C7822 Cyanothece sp. PCC 7822 ZP_03153783; C8802 Cyanothece sp. PCC 8802 ZP_03142797; Cagg Chloroflexus aggregans DSM 9485 YP_002461848; Caur10-fl Chloroflexus aurantiacus J-10-fl YP_001636362; CCY0110 Cyanothece sp. CCY 0110 ZP_01728928; Chyd Carboxydothermus hydrogenoformans Z-2901 YP_360377; Cjej1221 Campylobacter jejuni RM1221 YP_179388; Ckos895 Citrobacter koseri ATCC BAA-895 YP_001455880; Clim245 Chlorobium limicola DSM 245 YP_001942914; CmedTB-2 Caminibacter mediatlanticus TB-2 ZP_01871651; Cpha Chlorobium phaeobacteroides DSM 266 YP_911445; CtepTLS Chlorobium tepidum TLS NP_661672; Cwat8501 Crocosphaera watsonii WH 8501 ZP_00519188; Dbac Desulfomicrobium baculatum 1CC1_L; DBAV1 Dehalococcoides sp. BAV1 YP_001213724; Deth Dehalococcoides ethenogenes 195 YP_180861; DvulDP4 Desulfovibrio vulgaris DP4 YP_966691; Ecar1043 Pectobacterium atrosepticum SCRI1 [file pone.0013846.s005.doc]

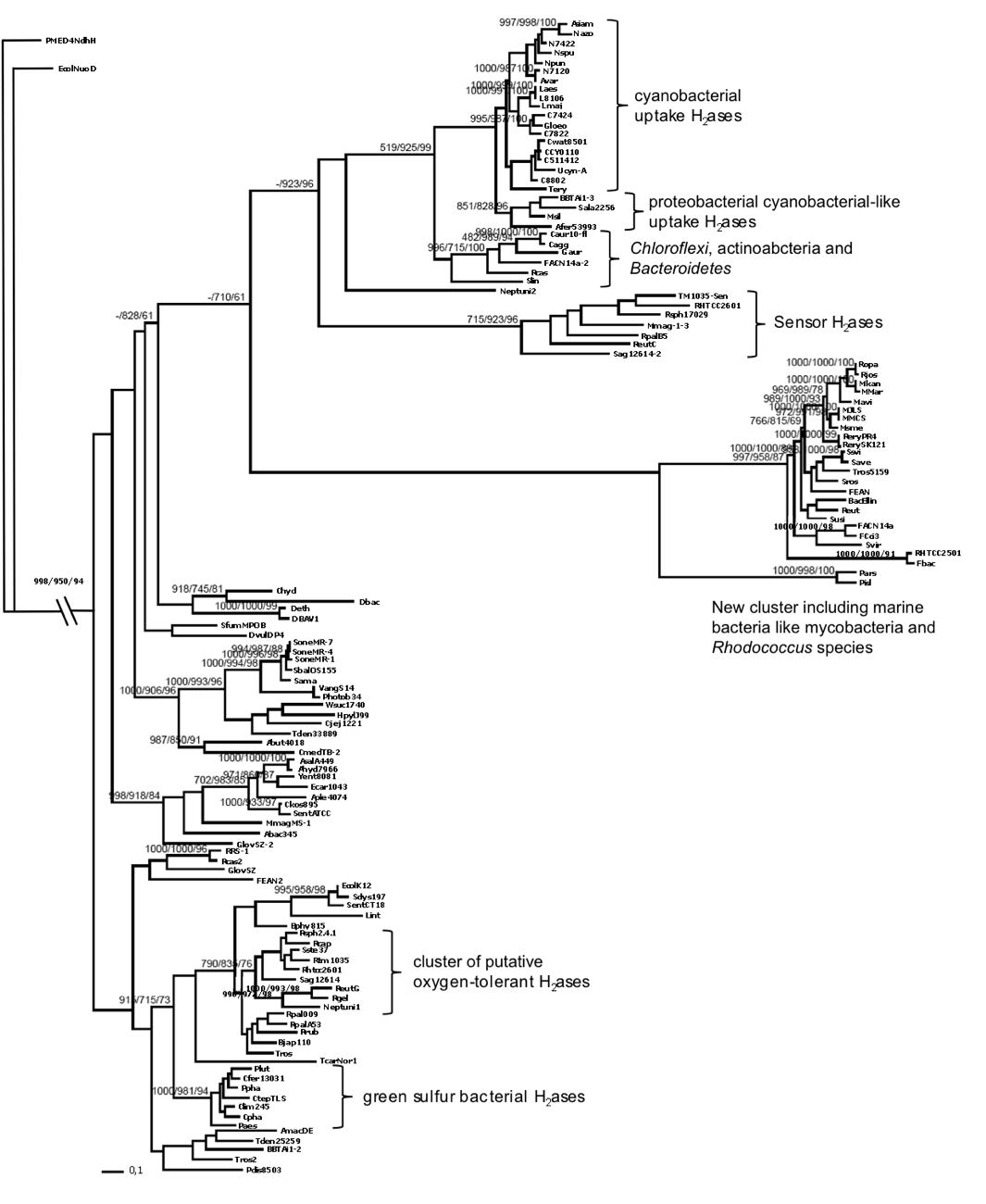


Fig. S4: Phylogenetic tree of HupL sequences. Representatives of the 49 kDa subunit of the complex I have been used as outgroup. The used abbreviations and their respective accession numbers are as follows: Abac345 *Candidatus Koribacter versatilis* Ellin345 YP_593314; Abut4018 *Arcobacter butzleri* RM4018 YP_001490358; Afer53993 *Acidithiobacillus ferrooxidans* ATCC 53993 YP_002219307; Ahyd7966 *Aeromonas hydrophila* subsp. *hydrophila* ATCC 7966 YP_857036; AmacDE *Alteromonas macleodii* 'Deep ecotype' YP_002124659; Aple4074 *Actinobacillus pleuropneumoniae* serovar 1 str. 4074 ZP_00134404; AsalA449 *Aeromonas salmonicida* subsp. *salmonicida* A449 YP_001141617; Asiam *Anabaena siamensis* TISTR 8012 AAN65266; Avar *Anabaena variabilis* ATCC 29413 YP_325087; Bac Ellin *bacterium Ellin514* ZP_03626632; BBTAi1-2 *Bradyrhizobium* sp. BTAi1 YP_001220511; BBTAi1-3 *Bradyrhizobium* sp. BTAi1 YP_001236652; Bjap110 *Bradyrhizobium japonicum* USDA 110 NP_773581; Bphy815 Burkholderia phymatum STM815 YP_001863308; C.fer13031 *Chlorobium ferrooxidans* DSM 13031 ZP_01386726; C511412 *Cyanothece* sp. ATCC 51142 YP_001802481; C7424 *Cyanothece* sp. PCC 7424 YP_002377118; C7822 Cyanothece sp. PCC 7822 ZP_03153783; C8802 Cyanothece sp. PCC 8802 ZP_03142797; Cagg *Chloroflexus aggregans* DSM 9485 YP_002461848; Caur10-fl *Chloroflexus aurantiacus* J-10-fl YP_001636362; CCY0110 *Cyanothece* sp. CCY 0110 ZP_01728928; Chyd *Carboxydothermus* *hydrogenoformans* Z-2901 YP_360377; Cjej1221 *Campylobacter jejuni* RM1221 YP_179388; Ckos895 *Citrobacter koseri* ATCC BAA-895 YP_001455880; Clim245 *Chlorobium limicola* DSM 245 YP_001942914; CmedTB-2 Caminibacter mediatlanticus TB-2 ZP_01871651; Cpha *Chlorobium phaeobacteroides* DSM 266 YP_911445; CtepTLS *Chlorobium tepidum* TLS NP_661672; Cwat8501 *Crocosphaera watsonii* WH 8501 ZP_00519188; Dbac Desulfomicrobium baculatum 1CC1_L; DBAV1 Dehalococcoides sp. BAV1 YP_001213724; Deth *Dehalococcoides ethenogenes* 195 YP_180861; DvulDP4 *Desulfovibrio vulgaris* DP4 YP_966691; Ecar1043 Pectobacterium atrosepticum SCRI1043 YP_049334; EcolK12 *Escherichia coli* str. K-12 substr. MG1655 NP_415492; EcolNuoD *Escherichia coli* CAA48363; FACN14a *Frankia alni* ACN14a YP_712616; FACN14a-2 *Frankia alni* ACN14a YP_712064; Fbac *Flavobacteria bacterium* MS024-2A ZP_03702421; FCci3 *Frankia* sp. CcI3 YP_481046; FEAN *Frankia* sp. EAN1pec YP_001506830; FEAN2 *Frankia* sp. EAN1pec YP_001507712; Gaur *Gemmatimonas aurantiaca* T-27 YP_002759924; Gloeo *Gloeothece* sp. PCC 6909 AAP04005; GlovSZ *Geobacter lovleyi* SZ YP_001952291; GlovSZ-2 *Geobacter lovleyi* SZ YP_001950403; HpylJ99 *Helicobacter pylori* J99 NP_223293; L8106 *Lyngbya* sp. PCC 8106 ZP_01619041; Laes *Lyngbya aestuarii* ABD34838; Lint *Lawsonia intracellularis* PHE/MN1-00 YP_594816; Lmaj *Lyngbya majuscula* CCAP 1446/4 AAO66476; Mavi *Mycobacterium avium* 104 YP_881873; MJLS *Mycobacterium* sp. JLS YP_00107040; Mkan *Mycobacterium kansasii* ATCC 12478 ZP_04750138; Mmag-1-3 *Magnetospirillum* *magneticum* AMB-1 YP_421305; MmagMS-1 *Magnetospirillum magnetotacticum* MS-1 ZP_00052632; Mmar *Mycobacterium marinum* M YP_001850173; MMCS *Mycobacterium* sp. MCS YP_639307; Msil *Methylocella silvestris* BL2 YP_002364007; Msme *Mycobacterium* *smegmatis* str. MC2 155 YP_887053; N7120 *Nostoc* sp. PCC 7120 NP_484720; N7422 *Nostoc* sp. PCC 7422 BAE46791; Nazo *'Nostoc azollae'* 0708 ZP_03768004; Neptuni2 *Neptuniibacter caesariensis* ZP_01167270; Neptuni 1 *Neptuniibacter caesariensis* ZP_01166595; Npun *Nostoc punctiforme* PCC 73102 AAC16277; Nspu *Nodularia spumigena* CCY 9414 ZP_01628406; Paes Prosthecochloris aestuarii DSM 271 YP_002015547; Pars *Pyrobaculum* arsenaticum DSM 13514 YP_001153513; Pdis8503 Parabacteroides distasonis ATCC 8503 YP_001303173; Photob34 *Photobacterium* sp. SKA34 ZP_01160131; Pisl *Pyrobaculum islandicum* DSM 4184 YP_929722; Plut *Pelodictyon luteolum* DSM 273 YP_375349; PMED4NdH *Prochlorococcus* *marinus* subsp. *pastoris* str. CCMP1986 NP_892293; Ppha *Pelodictyon phaeoclathratiforme* BU-1 YP_002018704; Rcap *Rhodobacter capsulatus* AAA69668; Rcas *Roseiflexus castenholzii* DSM 13941 YP_001433219; Rcas2 *Roseiflexus castenholzii* DSM 13941 YP_001433862; ReryPR4 *Rhodococcus erythropolis* PR4 YP_002766098; RerySK121 *Rhodococcus erythropolis* SK121 ZP_04384689; Reut *Ralstonia eutropha* H16 NP_942704; ReutC *Ralstonia eutropha* H16 NP_942663; ReutG *Ralstonia eutropha* H16 AAA16462; Rgel *Methylibium petroleiphilum* PM1 YP_001022015; RHTCC2501 *Robiginitalea biformata* HTCC2501 ZP_01119574; Rhtcc2601 *Roseovarius* sp. HTCC2601 ZP_01443057; RHTCC2601-Sens *Roseovarius* sp. HTCC2601 ZP_01443054; Rjos *Rhodococcus jostii* RHA1 YP_704548; Ropa *Rhodococcus opacus* B4 YP_002781742; Rpal009 *Rhodopseudomonas palustris* CGA009 NP_946314; RpalA53 *Rhodopseudomonas palustris* BisA53 YP_780164; RpalB5 *Rhodopseudomonas* *palustris* BisB5 YP_568300; RRS-1 *Roseiflexus* sp. RS-1 YP_001276649; Rrub *Rhodospirillum* *rubrum* ATCC 11170 YP_426250; Rsph17029 *Rhodobacter sphaeroides* ATCC 17029 YP_001044019; Rsph2.4.1 *Rhodobacter sphaeroides* 2.4.1 YP_353568; Rtm1035 *Roseovarius* sp. TM1035 ZP_01881109; Sag12614 *Stappia aggregata* IAM 12614 ZP_01550392; Sag12614-2 *Stappia aggregata* IAM 12614 ZP_01550270; Sala2256 *Sphingopyxis alaskensis* RB2256 YP_611130; Sama *Shewanella amazonensis* SB2B YP_927554; Save *Streptomyces avermitilis* MA-4680 NP_828543; SbalOS155 *Shewanella baltica* OS155 YP_001050263; Sdys197 *Shigella dysenteriae* Sd197 YP_402612; SentATCC *Salmonella enterica* subsp. *enterica serovar* *Paratyphi* A str. ATCC 9150 YP_152163; SentCT18 *Salmonella enterica* subsp. *enterica serovar* *Typhi* str. CT18 NP_456296; SfumMPOB *Syntrophobacter fumaroxidans* MPOB YP_847061; Slin *Spirosoma linguale* DSM 74 ZP_04492490; SoneMR-1 *Shewanella oneidensis* MR-1 NP_717701; SoneMR-4 *Shewanella* sp. MR-4 YP_733952; SoneMR-7 *Shewanella* sp. MR-7 YP_738201; Sros *Streptosporangium roseum* DSM 43021 ZP_04474993; Sste37 *Sagittula stellata* E-37 ZP_01748533; Ssvi *Streptomyces sviceus* ATCC 29083 YP_002204206; Susi *Solibacter usitatus* Ellin6076 YP_827763; Svir *Saccharomonospora* *viridis* DSM 43017 ZP_04507584; TcarNor1 *Thermosinus carboxydivorans* Nor1 ZP_01667576; Tden25259 *Thiobacillus denitrificans* ATCC 25259 YP_315133; Tden33889 *Sulfurimonas* *denitrificans* DSM 1251 YP_393947; Tery *Trichodesmium erythraeum* IMS101 YP_722943; TM1035-Sens *Roseovarius* sp. TM1035 ZP_01881113; Tros 5159 *Thermomicrobium roseum* DSM 5159 YP_002523076; Tros2 *Thiocapsa roseopersicina* AAA27410; Tros *Thiocapsa* *roseopersicina* AAC38282; Ucyn-A *Cyanothece* sp. CCY 0110 ZP_01728928; VangS14 *Vibrio angustum* S14 ZP_01234606; Wsuc1740 *Wolinella succinogenes* DSM 1740 NP_907813; Yent8081 *Yersinia* *enterocolitica* subsp. *enterocolitica* 8081 YP_001007767. The sequence of the marine unicellular group A cyanobacteria has been generated from the available short reads [70].
