# Supplementary material for: Distribution Analysis of Hydrogenases in Surface Waters of Marine and Freshwater Environments
Source: PLoS One. 2010 Nov 5;5(11):e13846. doi: 10.1371/journal.pone.0013846 (PMC2974642; doi:10.1371/journal.pone.0013846)
Supplement: Figure S5 — Distribution of small subunits of the bidirectional NAD(P)+ linked hydrogenase found in the GOS database of the different prokaryotic groups. The small subunit gene, hoxY, of Synechocystis has been used for the search. All genes have been retrieved form Punta Comorant, a hypersaline pond on the Galapagos Islands. (0.06 MB DOC) [file pone.0013846.s006.doc]

Fig. S5: Distribution of small subunits of the bidirectional NAD(P)^+^ linked hydrogenase found in the GOS database of the different prokaryotic groups. The small subunit gene, hoxY, of *Synechocystis* has been used for the search. All genes have been retrieved form Punta Comorant, a hypersaline pond on the Galapagos Islands.
