# Supplementary material for: Distribution Analysis of Hydrogenases in Surface Waters of Marine and Freshwater Environments
Source: PLoS One. 2010 Nov 5;5(11):e13846. doi: 10.1371/journal.pone.0013846 (PMC2974642; doi:10.1371/journal.pone.0013846)
Supplement: Figure S6 — Distribution of small subunits of the membrane bound H2 uptake hydrogenasses found in the GOS database of the different prokaryotic groups. The hupS sequence of Desulfovibrio vulgaris was used for the search. On the right the number of sequences from the different sampling stations is shown. (0.08 MB DOC) [file pone.0013846.s007.doc]

Fig. S6: Distribution of small subunits of the membrane bound H_2_ uptake hydrogenasses found in the GOS database of the different prokaryotic groups. The *hupS* sequence of *Desulfovibrio vulgaris* was used for the search. On the right the number of sequences from the different sampling stations is shown.
