# Supplementary material for: Distribution Analysis of Hydrogenases in Surface Waters of Marine and Freshwater Environments
Source: PLoS One. 2010 Nov 5;5(11):e13846. doi: 10.1371/journal.pone.0013846 (PMC2974642; doi:10.1371/journal.pone.0013846)
Supplement: Figure S7 — Distribution of small subunits of the cyanobacterial-like uptake hydrogenase found in the GOS database of the different prokaryotic groups. The small subunit gene, hupS, of Nostoc sp. PCC 7120 has been used for the search. (0.05 MB DOC) [file pone.0013846.s008.doc]

Fig. S7: Distribution of small subunits of the cyanobacterial-like uptake hydrogenase found in the GOS database of the different prokaryotic groups. The small subunit gene, *hupS*, of *Nostoc* sp. PCC 7120 has been used for the sear
