# Supplementary material for: Distribution Analysis of Hydrogenases in Surface Waters of Marine and Freshwater Environments
Source: PLoS One. 2010 Nov 5;5(11):e13846. doi: 10.1371/journal.pone.0013846 (PMC2974642; doi:10.1371/journal.pone.0013846)
Supplement: Figure S8 — Phylogenetic tree of HoxH sequences. Representatives of the 49 kDa subunit of the complex I have been used as outgroup. The used abbreviations and their respective accession numbers are as follows: Afla Acetomicrobium flavidum CAA56464; Ahalo Aphanothce halophytica GQ454444; Amar Acaryochloris marina MBIC11017 YP_001521996; Amax Arthrospira maxima FACHBSM AAQ63961; Apla1 Arthrospira platensis FACHB341 AAQ63964; Apla2 Arthrospira platensis FACHBOUQDS6 AAQ63959; Apla3 Arthrospira platensis FACHB439 AAQ63960; Apla4 Arthrospira platensis FACHB791 AAQ91344; Avar Anabaena variabilis ATCC 29413 YP_325153; Bxen Burkholderia xenovorans LB400 YP_555781; Cagg Chloroflexus aggregans DSM 9485 YP_002463784; CaggL Chlorobium chlorochromatii CaD3 YP_378564; Caur Chloroflexus aurantiacus J-10-fl YP_001634807; CCY0110 Cyanothece sp. CCY 0110 ZP_01727423; ClimL Chlorobium limicola DSM 245 YP_001944104; Cnec Ralstonia eutropha H16 NP_942730; CphaL Chlorobium phaeobacteroides DSM 266 YP_912598;CtepL Chlorobium tepidum TLS NP_662771; Daro Dechloromonas aromatica RCB YP_284208; DethV Dehalococcoides ethenogenes 195 YP_181357; Dpsy Desulfotalea psychrophila LSv54 YP_065948; DpsyV Desulfotalea psychrophila LSv54 YP_064749;Ecol Escherichia coli CAA48363; Galp Gloeocapsa alpicola str. CALU 743 AAO85440; Gmet1 Geobacter metallireducens GS-15 YP_384078; Gmet2 Geobacter metallireducens GS-15 YP_386258; GOS1 and GOS2 are the two consenus sequeces retrieved from the GOS database; Gsul1 Geobacter sulfurreducens PCA NP_953465; Gsul2 Geobacter sulfurreducens PCA NP_953763; Lyng Lyngbya majuscula CCAP 1446/4 AAT07678; Magneto Magnetococcus sp. MC-1 YP_864809; Mastigo Mastigocladus laminosus SAG 4.84 GQ454445; Mcap Methylococcus capsulatus str. Bath YP_112653; MferV Methanothermus fervidus Q49179; MjanV Methanocaldococcus jannaschii DSM 2661 NP_248187; Mkan Methanopyrus kandleri AV19 NP_613553; Mmag Magnetospirillum magnetotacticum MS-1 ZP_00053777; MmarV Methanococcus maripaludis S2 NP_987943;MvolV1 M [file pone.0013846.s009.doc]

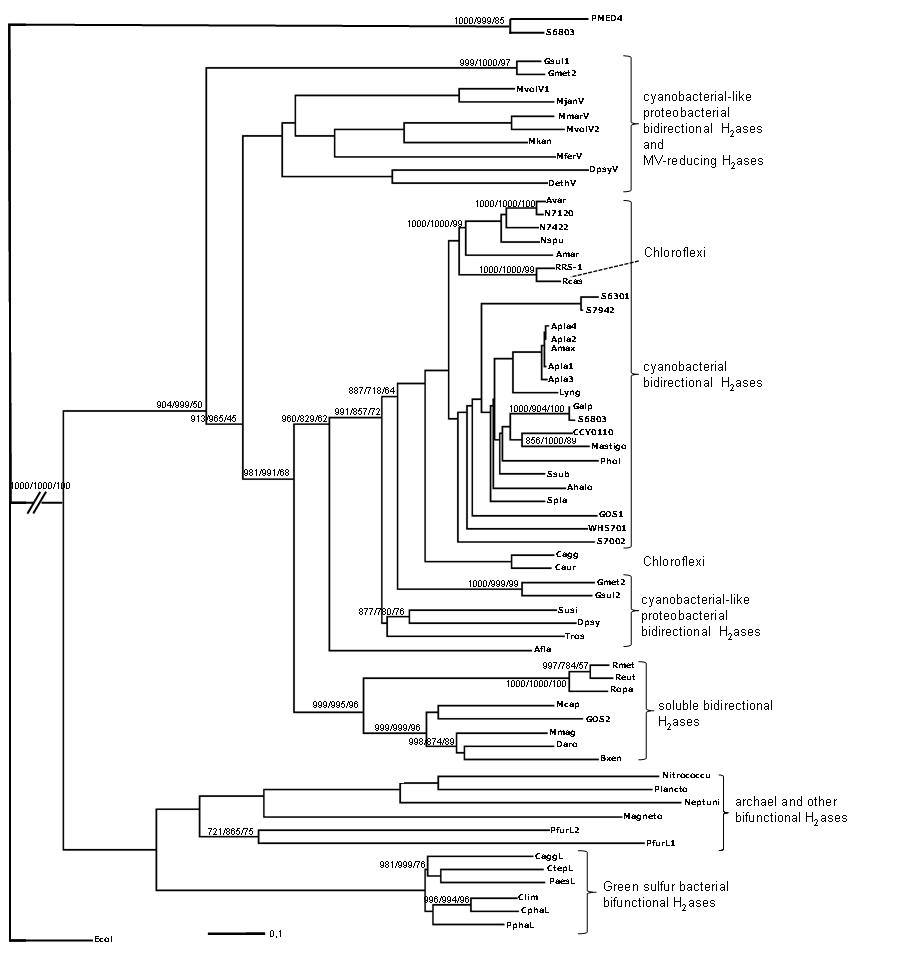


Fig. S8: Phylogenetic tree of HoxH sequences. Representatives of the 49 kDa subunit of the complex I have been used as outgroup. The used abbreviations and their respective accession numbers are as follows: Afla *Acetomicrobium flavidum* CAA56464; Ahalo *Aphanothce halophytica* GQ454444; Amar *Acaryochloris marina* MBIC11017 YP_001521996; Amax *Arthrospira maxima* FACHBSM AAQ63961; Apla1 *Arthrospira platensis* FACHB341 AAQ63964; Apla2 *Arthrospira platensis* FACHBOUQDS6 AAQ63959; Apla3 *Arthrospira platensis* FACHB439 AAQ63960; Apla4 *Arthrospira platensis* FACHB791 AAQ91344; Avar *Anabaena variabilis* ATCC 29413 YP_325153; Bxen *Burkholderia xenovorans* LB400 YP_555781; Cagg *Chloroflexus aggregans* DSM 9485 YP_002463784; CaggL *Chlorobium chlorochromatii* CaD3 YP_378564; Caur *Chloroflexus aurantiacus* J-10-fl YP_001634807; CCY0110 *Cyanothece* sp. CCY 0110 ZP_01727423; ClimL *Chlorobium limicola* DSM 245 YP_001944104; Cnec *Ralstonia eutropha* H16 NP_942730; CphaL *Chlorobium phaeobacteroides* DSM 266 YP_912598;CtepL *Chlorobium tepidum* TLS NP_662771; Daro *Dechloromonas aromatica* RCB YP_284208; DethV *Dehalococcoides ethenogenes* 195 YP_181357; Dpsy *Desulfotalea psychrophila* LSv54 YP_065948; DpsyV *Desulfotalea psychrophila* LSv54 YP_064749;Ecol *Escherichia coli* CAA48363; Galp *Gloeocapsa alpicola* str. CALU 743 AAO85440; Gmet1 *Geobacter metallireducens* GS-15 YP_384078; Gmet2 *Geobacter metallireducens* GS-15 YP_386258; GOS1 and GOS2 are the two consenus sequeces retrieved from the GOS database; Gsul1 *Geobacter sulfurreducens* PCA NP_953465; Gsul2 *Geobacter sulfurreducens* PCA NP_953763; Lyng *Lyngbya majuscula* CCAP 1446/4 AAT07678; Magneto *Magnetococcus* sp. MC-1 YP_864809; Mastigo *Mastigocladus laminosus* SAG 4.84 GQ454445; Mcap *Methylococcus capsulatus* str. Bath YP_112653; MferV *Methanothermus fervidus* Q49179; MjanV *Methanocaldococcus jannaschii* DSM 2661 NP_248187; Mkan *Methanopyrus kandleri* AV19 NP_613553; Mmag *Magnetospirillum magnetotacticum* MS-1 ZP_00053777; MmarV *Methanococcus maripaludis* S2 NP_987943;MvolV1 *Methanococcus voltae* Q00404; MvolV2 *Methanococcus voltae* Q00407;N7120 *Nostoc* sp. PCC 7120 NP_484809; N7422 *Nostoc* sp. PCC 7422 BAE46796; Neptuni *Oceanospirillum* sp. MED92 ZP_01164927; Nitrococcus *Nitrococcus mobilis* Nb-231 ZP_01126922; Nspu *Nodularia spumigena* CCY 9414 ZP_01629499; Nspu *Nodularia spumigena* CCY 9414 ZP_01629499; PaesL *Prosthecochloris aestuarii* DSM 271 YP_002016588; PfurL1 *Pyrococcus furiosus* DSM 3638 NP_578623; PfurL2 *Pyrococcus furiosus* DSM 3638 NP_579061; Phol *Prochlorothrix hollandica* AAB53705;Plancto *Planctomyces maris* DSM 8797 ZP_01852867; PMED4 *Prochlorococcus marinus* subsp. *pastoris* str. CCMP1986 NP_892293; PphaL *Pelodictyon phaeoclathratiforme* BU-1 YP_002019299; Rcas *Roseiflexus castenholzii* DSM 13941 YP_001431482; Rmet *Ralstonia metallidurans* CH34 YP_583677; Ropa *Rhodococcus opacus* AAB57892; RRS-1 *Roseiflexus* sp. RS-1 YP_001277847; S6301 *Synechococcus elongatus* PCC 6301 YP_172265; S6803 *Synechocystis* sp. PCC 6803 NP_441259; S6803 *Synechocystis* sp. PCC 6803 NP_441411;S7002 *Synechococcus* sp. PCC 7002 YP_001733469; S7942 *Synechococcus elongatus* PCC 7942 YP_401572; Spla *Arthrospira platensis* FACHB440 AAQ63963; Ssub *Spirulina subsalsa* FACHB351 AAQ63962; Susi *Solibacter usitatus* Ellin6076 YP_826256;Tros *Thiocapsa roseopersicina* AAP50523; WH5701 *Synechococcus* sp. WH 5701 ZP_01085930;
